# Multi-chaotic signal identification employing a causal cross-correlation neural network

**DOI:** 10.1016/j.isci.2026.116164

**Published:** 2026-06-26

**Authors:** Bingrui Wang, Xinyang Piao, Chengbin Chen, Ente Guo, Xingang Zhang

**Affiliations:** 1 Henan Collaborative Innovation Center of Intelligent Explosion-proof Equipment, Nanyang Normal University, Nanyang 473061, China; 2 Electrical Engineering Department, Columbia University, New York, NY 10027, USA; 3 College of Physics and Information Engineering, Fuzhou University, Fuzhou 350108, China; 4 School of Computer and Big Data, Minjiang University, Fuzhou 350108, China

**Keywords:** bioinformatics, neural networks, complex systems

## Abstract

Chaos identification plays a crucial role in comprehending complex systems. However, current methods face three challenges: neglect temporal causality, lack robustness against noise, and identify a limited number of chaotic signals. To address the challenges, we focus on identifying multi-chaotic signals with noise interference. First, this study examines 15 types of chaos models. We propose an acquisition-enhanced Bayesian optimization to improve the Swish-modulo map, which is then verified using the maximum Lyapunov exponent. Second, we present a causal cross-correlation network for chaos identification, which incorporates a learnable wavelet transform, time-frequency feature fusion, and spectral-inversion-free fast Fourier transform-based cross-correlation (SFFT-CC). Consequently, experimental results show that the chaos identification accuracy reaches 96.67% at a 20 dB noise. The SFFT-CC outperforms the traditional dilated convolution by up to 5.42% in chaos identification accuracy, while achieving a 43-fold reduction in training time. Theoretical analyses are provided to elucidate these results.

## Highlights

• Causal cross-correlation network identifies multi-chaotic signals in noisy data
• Attention-based fusion limits the Lipschitz constant, boosting noise robustness
• SFFT-CC preserves physical causality and vastly reduces multiplication operations
• Lightweight structure achieves high generalizability on unseen and real ECG data

## Graphical abstract

**Figure:**
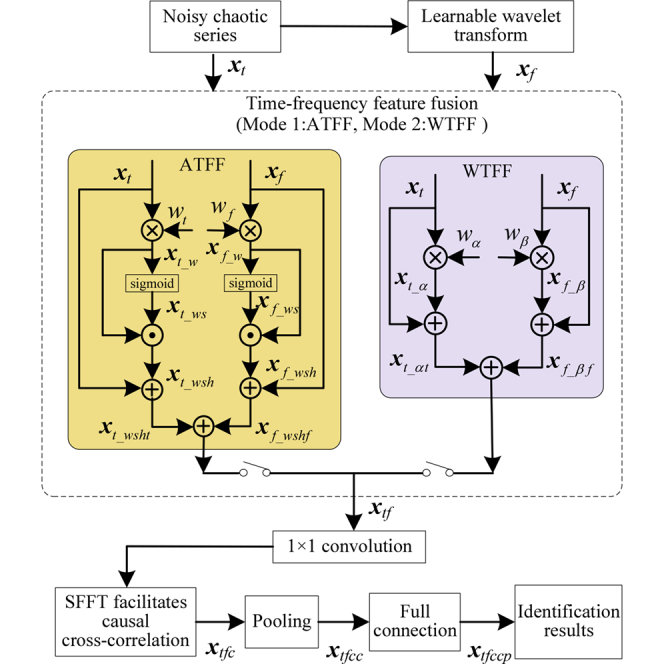

## Introduction

The accurate identification of chaotic dynamics is crucial in many critical fields. In epidemiology, determining whether the spread of a disease exhibits chaotic behavior helps formulate effective intervention strategies. In neuroscience, the question is whether the chaotic characteristics of brain activity are essential for understanding information-processing mechanisms. Furthermore, in fields such as biomedical engineering (e.g., heart rate analysis), engineering safety (e.g., mechanical fault diagnosis), and meteorological forecasting, it is vital to correctly determine whether an observed time series arises from chaotic dynamics to properly analyze the underlying system.^1^ This study focuses specifically on identifying multiple chaotic signals, thereby facilitating more informed and accurate decision-making.

Several deep learning methods have been proposed for the identification of chaotic sequences. Zanin^2^ implemented numerical experiments to evaluate the performance of different deep learning models in discriminating between stochastic and chaotic time series. Their results showed that deep learning outperformed other methods in terms of minimum time-series length and noise resilience. However, the study relied on existing deep learning models. Barrio et al.^3^ explored new deep learning techniques to solve chaos detection problems, focusing on two classical time series: the logistic map and the Lorenz system. They trained three types of networks (multi-layer perceptron, convolutional neural network [CNN], and long short-term memory cell) to classify the time series as regular or chaotic. The input data length for each group in their experiment reached 1,000.

Moreover, Lee and Flach^4^ trained an artificial neural network to distinguish between chaotic and regular dynamics in the two-dimensional (2D) Chirikov standard map. The performance of this neural network was evaluated against traditional methods using finite-length trajectories, demonstrating its superior accuracy over short-time scales. Furthermore, the neural network was successfully tested on the one-dimensional (1D) logistic map and the Lorenz system. Boullé et al.^5^ used standard deep neural networks to classify univariate time series generated by discrete and continuous dynamical systems based on their chaotic or non-chaotic behavior. They trained these networks on low-dimensional phase spaces, such as the logistic map, the sine-circle map, the Lorenz system, and the Kuramoto-Sivashinsky equation, demonstrating the generalizability of their approach. Galarza and Oraby^6^ applied a CNN to estimate the Lyapunov exponent using the prototype of the chaotic Lorenz system. By using 10,000 test curves, the CNN was approximately 600 times faster than the MATLAB method.

Recently, chaotic sequences were transformed into graphical representations. Zhou et al.^7^ demonstrated that the recurrent plot can transform the time series into a 2D image. The recurrent plots represent three major time series classes—chaotic, periodic, and random—generated by classical dynamics. These plots were then used as the dataset to train the residual neural network (ResNet). The precision of chaos identification could reach 97.6%. Different encoding methods and classification networks were compared in their experiments. The graphical images of time series from different chaotic systems were classified using deep learning methods.^8^ They generated a dataset of images from the time series of Chen and Rössler chaotic systems and applied transfer learning methods, including SqueezeNet, VGG-19, AlexNet, ResNet50, ResNet-101, DenseNet-201, ShuffleNet, and GoogLeNet. The classification accuracy ranged from 89% to 99.7%. However, the experiment required converting sequential data into images. Giuseppi et al.^9^ proposed a computationally efficient multimodal deep neural network by combining information from time series, recurrence plots, and spectrograms. The network was designed to determine whether a series of measurements was generated by chaotic dynamics.

Although existing studies have applied deep learning to chaos identification, most use networks designed for image processing (such as VGG-19 and ResNet) and neglect the intrinsic characteristics of chaotic sequences. Additionally, some studies convert sequences into images, thereby increasing computational complexity and neglecting the causal relationship. The length of input data sequences can reach 1,000, leading to a significant increase in computational operations. Furthermore, some studies also lack analysis of mixed sequences with superimposed noise and cover only a limited range of chaos types. To address these issues, this work proposes a deep learning model for causal computation that extracts both time-domain and frequency-domain features from chaotic sequences and incorporates various types of noise. We expect the model to adapt to different noise conditions, identify a wide range of chaotic sequences, and improve the accuracy of chaos identification.

## Results

### The proposed causal cross-correlation neural network

**Figure 1:**
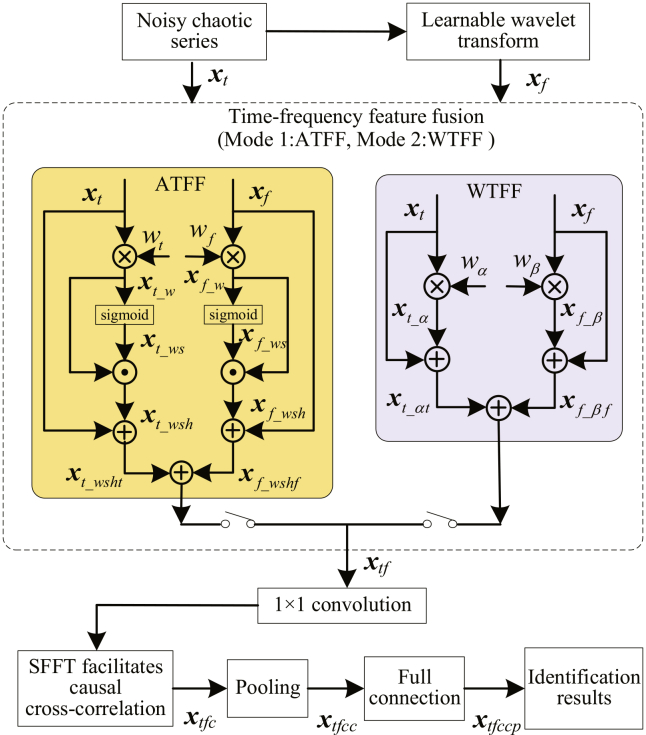
The overall neural network

In Figure 1, the input sequence ***x***_*t*_ is a chaotic sequence affected by noise. A learnable wavelet transform module extracts frequency features ***x***_*f*_ from the chaotic sequence ***x***_*t*_. These time-frequency features are then fused, yielding the fused features ***x***_*tf*_. Specifically, we propose two time-frequency feature fusion modes: attention-based time-frequency fusion (ATFF) and weighted time-frequency fusion (WTFF). Detailed structural variables and intermediate features (e.g., ***x***_*t*_*w*_, ***x***_*f*_*w*_) within these modes are thoroughly discussed in the discussion section. A 1 × 1 convolution is applied to refine the fused features ***x***_*tf*_, yielding ***x***_*tfc*_. We propose a spectral-inversion-free fast Fourier transform (SFFT) that facilitates causal cross-correlation, further processes the features ***x***_*tfc*_, and labels the result as ***x***_*tfcc*_. Then, a pooling module is utilized to compress the parameters. The pooled result is denoted as ***x***_*tfccp*_. Finally, a fully connected module provides the identification outcome.

### Experimental results

#### Experimental setup and parameter settings

We utilize 15 chaotic equations (introduced in STAR Methods) as the data source. The dataset contains 15 types of chaotic sequences, each with 160,000 data points, for a total of 2.4 million data points. After assembling the dataset, we split it into training and test sets in an 8:2 ratio. To mimic real-world noise, we then add noise at five signal-to-noise ratios (SNRs): 20, 17.5, 15, 12.5, and 10 dB. The model is subsequently trained using the structure shown in Figure 1, with a batch size of 128 for 100 iterations. To speed up evaluation of chaotic identification accuracy, we select 15 of the 100 trained models—specifically, the 30th, 35th, 40th, 45th, 50th, 55th, 60th, 65th, 70th, 75th, 80th, 85th, 90th, 95th, and 100th. We evaluate these 15 models on the test set and record the maximum identification accuracy.

For pooling operations, 1 × 2 average pooling computes the mean of two consecutive data points, while 1 × 8 average pooling computes the mean over eight. For simplicity, we refer to 1 × 2 and 1 × 8 average pooling as AvgP-2 and AvgP-8, respectively. We next detail the ablation study design. Based on the structure in Figure 1, we propose six variants, summarized in Table 1. These six variants all contain 1 × 1 convolution, a spectral-inversion-free FFT-based cross-correlation (SFFT-CC), and a full connection module. In structure 1, the fusion module from Figure 1 is replaced with ATFF, and the pooling operation uses AvgP-8. All other modules remain unchanged. Therefore, the sequence of modules in structure 1 is: wavelet module, ATFF, 1 × 1 convolution, SFFT-CC, AvgP-8, and a full connection module. Structure 1 is referred to as ATFF with AvgP-8 (ATFF-P8). Notably, in structures 5 and 6, both the wavelet transform module and the fusion module are removed. Structure 5 is abbreviated non-WT-P8, and structure 6 is non-WT-P2.

**Table 1:** The six structures

	Wavelet module included?	Fusion approach	Pooling method	Structure name and abbreviation
Structure 1	yes	ATFF	avg P-8	ATFF with avg P-8 (ATFF-P8)
Structure 2	yes	WTFF	avg P-8	WTFF with avg P-8 (WTFF-P8)
Structure 3	yes	ATFF	avg P-2	ATFF with avg P-2 (ATFF-P2)
Structure 4	yes	WTFF	avg P-2	WTFF with avg P-2 (WTFF-P2)
Structure 5	no	not applicable	avg P-8	non-wavelet transform with AvgP-8 (non-WT-P8)
Structure 6	no	not applicable	avg P-2	non-wavelet transform with AvgP-2 (non-WT-P2)

#### Robustness analysis

We assess the impact of these six structures across five SNRs. Table 2 shows the model accuracy for each structure, and parameter counts are order-of-magnitude estimates.

**Table 2:** Accuracy and parameter count of the six structures under five SNRs

	SNR (dB)	ATFF-P8	WTFF-P8	Non-WT-P8	ATFF-P2	WTFF-P2	Non-WT-P2
Accuracy	20	96.67%	96.35%	95.64%	96.02%	95.70%	95.36%
	17.5	94.47%	93.93%	93.53%	93.41%	92.92%	93.27%
	15	89.37%	89.00%	88.50%	88.47%	88.32%	88.19%
	12.5	83.63%	83.63%	82.83%	82.81%	82.73%	82.09%
	10	76.43%	76.36%	75.01%	75.11%	74.99%	74.05%
Magnitude of parameters		2 × 10^3^	2 × 10^3^	2 × 10^3^	7 × 10^3^	7 × 10^3^	7 × 10^3^

To clarify the results, we compare the accuracy of six structures in Table 3. The table shows that, across various noise levels, the ATFF and WTFF with wavelet structures generally perform better than the non-WT-P8 and non-WT-P2 without wavelets.

**Table 3:** Detailed accuracy comparison of the six structures

SNR (dB)	ATFF-P8 vs. non-WT-P8	WTFF-P8 vs. non-WT-P8	ATFF-P2 vs. non-WT-P2	WTFF-P2 vs. non-WT-P2	ATFF-P8 vs. non-WT-P2	WTFF-P8 vs. non-WT-P2	Non-WT-P8 vs. non-WT-P2
20	1.03%	0.71%	0.66%	0.34%	1.31%	0.99%	0.28%
17.5	0.94%	0.40%	0.14%	−0.35%	1.20%	0.66%	0.26%
15	0.87%	0.50%	0.28%	0.13%	1.18%	0.81%	0.31%
12.5	0.80%	0.80%	0.72%	0.64%	1.54%	1.54%	0.74%
10	1.42%	1.35%	1.06%	0.94%	2.38%	2.31%	0.96%

For a more specific illustration, consider 20 dB SNR. The following observations are made: (1) ATFF-P8 and WTFF-P8 improve accuracy by 1.03% and 0.71% over non-WT-P8. (2) ATFF-P2 and WTFF-P2 gain 0.66% and 0.34% compared to non-WT-P2. (3) Relative to non-WT-P2, ATFF-P8 and WTFF-P8 increase by 1.31% and 0.99%. (4) Non-WT-P8 exceeds non-WT-P2 by 0.28%. Even at a lower SNR of 10 dB, the wavelet module remains effective: ATFF-P8 and WTFF-P8 outperform non-WT-P2 by 2.38% and 2.31%, respectively.

To further evaluate the applicability of the proposed method on real-world chaotic data, an additional experiment is conducted using the publicly available PTB Diagnostic ECG Database.^10^ Electrocardiogram signals are widely considered to possess chaotic dynamical characteristics. In this task, we employ the non-WT-P8 network to classify myocardial infarction and normal heartbeats. To maintain consistency, the training configurations, such as the number of epochs, are kept identical to those used in the 15 chaos identification task. The non-WT-P8 network achieves a classification accuracy of 92.47% on this real-world dataset. Although the causal non-WT-P8 model relies on no future information and maintains a small parameter magnitude of approximately 2 × 10^3^, achieving such high accuracy indicates the practical robustness of the proposed architecture.

#### Generalizability analysis

A fundamental property of chaotic systems is their sensitivity to initial conditions. For a given chaotic map, a slight change in its initial value (*x*_0_) usually generates a divergent sequence trajectory. In the preceding identification tasks, the chaotic sequences are evaluated under the condition that both the training and testing sets shared the same initial value. To further assess the generalizability of the proposed networks, we design an additional testing scenario: the models, initially trained on sequences generated with *x*_0_, are subsequently evaluated on new sequences generated with a different initial value (*x*∗).

Table 4 summarizes the identification accuracies of the ATFF-P8 and non-WT-P8 architectures under varying SNRs. For clarity, the “same *x*_0_” columns denote the baseline performance, where the training and testing data share the same initial condition, while the “new *x*∗” columns indicate the test performance on the newly generated sequence trajectories. The accuracy drop between these two conditions is denoted as “drop (*↓*).”

**Table 4:** Identification accuracy (%) of the networks under various SNR levels for different initial values

SNR (dB)	ATFF-P8			Non-WT-P8		
	same *x*_0_	new *x*∗	drop (*↓*)	same *x*_0_	new *x*∗	drop (*↓*)
20	96.67	96.667	0.003	95.64	95.607	0.033
17.5	94.47	94.427	0.043	93.53	93.527	0.003
15	89.37	89.340	0.030	88.50	88.487	0.013
12.5	83.63	83.573	0.057	82.83	82.780	0.050
10	76.43	76.267	0.163	75.01	74.947	0.063

As shown in Table 4, both networks maintain stable generalizability. When evaluated on the new sequences (*x*∗), the classification accuracies experience a slight decrease. For instance, at an SNR of 20 dB, the accuracy drop for ATFF-P8 is 0.003%, and for non-WT-P8, it is 0.033%. Under the 10 dB SNR condition, the performance degradation remains relatively small (0.163% for ATFF-P8 and 0.063% for non-WT-P8).

These consistent results suggest that the proposed architectures do not merely memorize the specific time-domain waveforms of the training sequences. Instead, they appear to capture the underlying dynamical characteristics of the distinct chaotic systems. Consequently, the networks can maintain effective classification performance even when processing new data trajectories.

## Discussion

### Theoretical properties of ATFF and WTFF

#### Two time-frequency feature fusion structures

**Figure 2:**
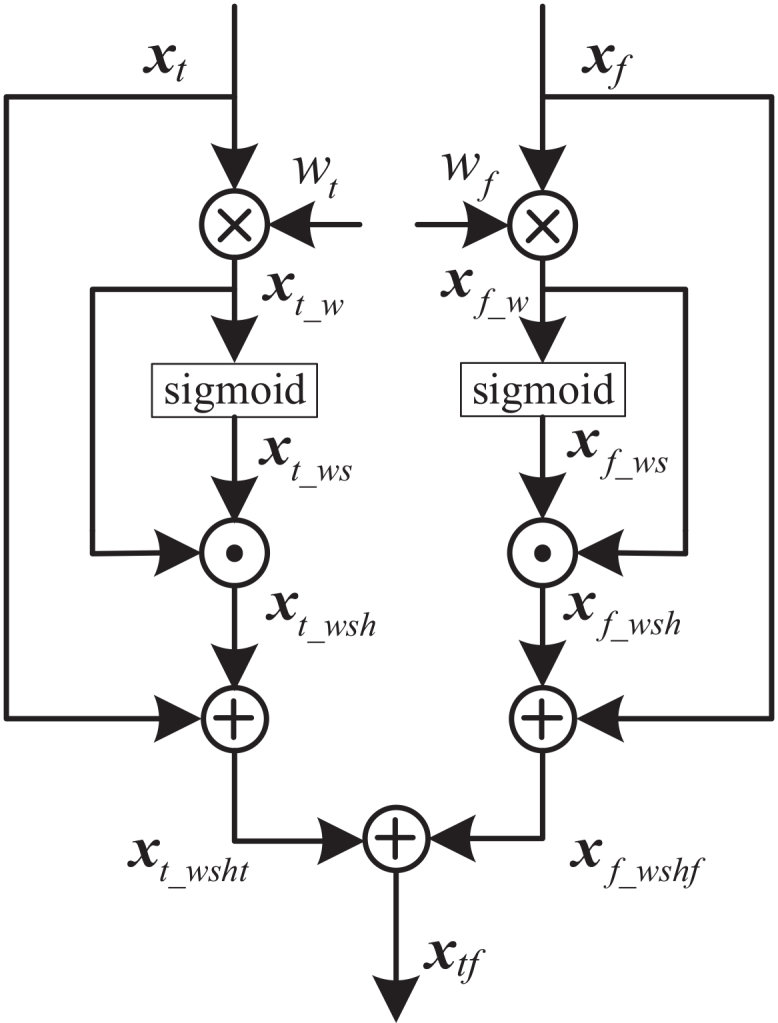
The structure of ATFF

Targeting the time-frequency domain fusion module depicted in Figure 1, we propose two different fusion structures. The first fusion structure for ***x***_*t*_ and ***x***_*f*_ is called ATFF, as shown in Figure 2. First, ***x***_*t*_ is multiplied by the weight parameter *w*_*t*_ to get ***x***_*t*_*w*_ and ***x***_*f*_ is multiplied by the weight parameter *w*_*f*_ to obtain ***x***_*f*_*w*_. Then, ***x***_*t*_*w*_ and ***x***_*f*_*w*_ undergo the sigmoid function to produce ***x***_*t*_*ws*_ and ***x***_*f*_*ws*_, respectively. Next, ***x***_*t*_*ws*_ and ***x***_*f*_*ws*_ are element-wise multiplied by ***x***_*t*_*w*_ and ***x***_*f*_*w*_, respectively to produce ***x***_*t*_*wsh*_ and ***x***_*f*_*wsh*_, respectively. The sigmoid function possesses gating properties. For example, when ***x***_*t*_*ws*_ is multiplied by ***x***_*t*_*w*_, some values of ***x***_*t*_*w*_ become smaller and less noticeable, while other values are highlighted. This represents an attention operation that focuses more on the dominant values in ***x***_*t*_*ws*_. To address the vanishing gradient issue caused by the small values of ***x***_*t*_*wsh*_ and ***x***_*f*_*wsh*_, ***x***_*t*_*wsh*_, and ***x***_*f*_*wsh*_ are added to ***x***_*t*_ and ***x***_*f*_ to obtain ***x***_*t*_*wsht*_ and ***x***_*f*_*wshf*_, respectively. Finally, ***x***_*t*_*wsht*_ and ***x***_*f*_*wshf*_ are summed to produce the fused output $x_{t_{f}}$, which can be expressed as

(Equation 1) $$\left\{\begin{array}{cc} x_{t\text{\_}w} & =w_{t}x_{t},\\ x_{f\text{\_}w} & =w_{f}x_{f},\\ sig\left(z\right) & =sig\left(z\right),\\ x_{t\text{\_}wsh} & =sig\left(x_{t\text{\_}w}\right)⊙x_{t\text{\_}w},\\ x_{f\text{\_}wsh} & =sig\left(x_{f\text{\_}w}\right)⊙x_{f\text{\_}w},\\ x_{tf} & =x_{t\text{\_}wsh}+x_{f\text{\_}wsh}+x_{t}+x_{f}\\ & =sig\left(w_{t}x_{t}\right)⊙\left(w_{t}x_{t}\right)+sig\left(w_{f}x_{f}\right)⊙\left(w_{f}x_{f}\right)+x_{t}+x_{f}. \end{array}\right)$$

**Figure 3:**
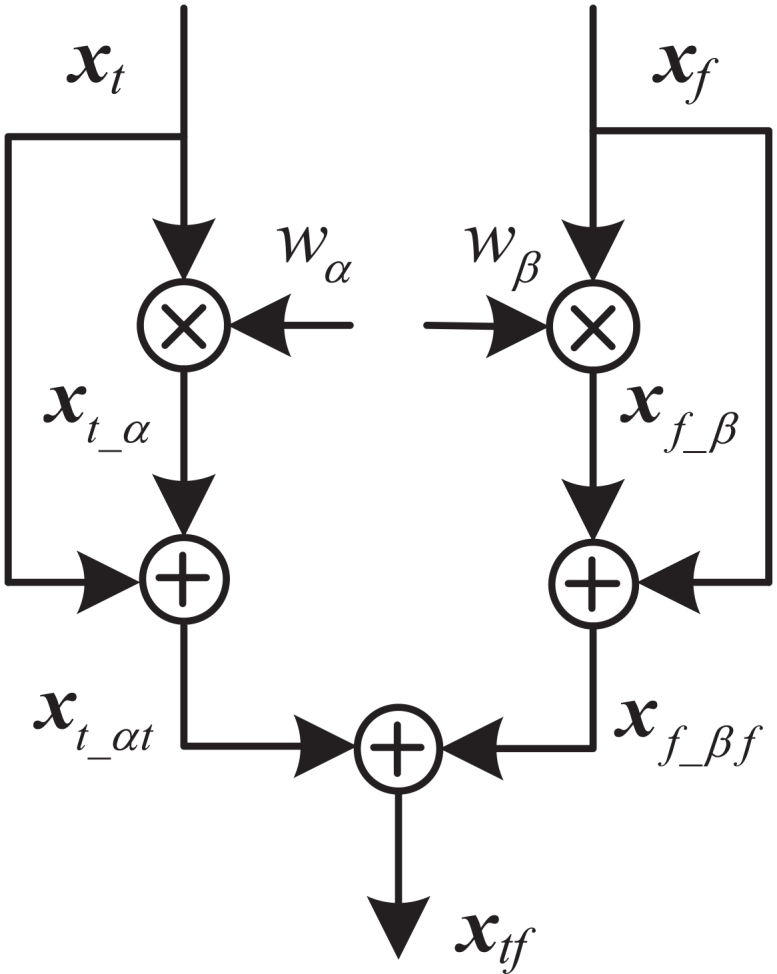
The structure of WTFF

The second fusion structure is WTFF, as shown in Figure 3. We multiply ***x***_*t*_ by a weight parameter *w*_*α*_ to obtain ***x***_*t*_*α*_, and similarly, ***x***_*f*_ by another weight *w*_*β*_ to obtain ***x***_*f*_*β*_. Since ***x***_*t*_ and ***x***_*f*_ are generated and transformed from chaotic sequences, their absolute values are generally less than 1, making the products ***x***_*t*_*α*_ and ***x***_*f*_*β*_ typically small. To address this, we add ***x***_*t*_*α*_ to the original ***x***_*t*_ to get ***x***_*t*_*αt*_ and ***x***_*f*_*β*_ to the original ***x***_*f*_ to get ***x***_*f*_*βf*_. Finally, we sum ***x***_*t*_*αt*_ and ***x***_*f*_*βf*_ to obtain the fused output $x_{t_{f}}$, given by:

(Equation 2) $$\left\{\begin{array}{cc} & x_{t\text{\_}\alpha }=w_{\alpha }x_{t},\\ & x_{f\text{\_}\beta }=w_{\beta }x_{f},\\ & x_{t\text{\_}\alpha t}=x_{t\text{\_}\alpha }+x_{t},\\ & x_{f\text{\_}\beta f}=x_{f\text{\_}\beta }+x_{f},\\ & x_{tf}=x_{t\text{\_}\alpha t}+x_{f\text{\_}\beta f}. \end{array}\right)$$

#### Lipschitz constants via theoretical analysis

We evaluate the representation stability of the ATFF and WTFF modules by analytically examining their Lipschitz continuity.

Definition 1: let *f*(***x***) be a continuously differentiable function. For any two points, ***x***_1_ and ***x***_2_ in its domain, *f*(***x***) is Lipschitz continuous if the Euclidean distance between their functional outputs is bounded by a proportional constant *K*:

(Equation 3) $${\left\|f\left(x_{1}\right)-f\left(x_{2}\right)\right\|}_{2}\leq K{\left\|x_{1}-x_{2}\right\|}_{2},$$

where ${\left\|\cdot \right\|}_{2}$ denotes the 2-norm.^11^^,^^12^ The minimal *K* satisfying Equation 3 is defined as the Lipschitz constant, denoted by *k*_0_.

By introducing a perturbation vector ***ξ*** = ***x***_1_ − ***x***_2_, Equation 3 can be equivalently expressed as:

(Equation 4) $${\left\|f\left(x+\xi \right)-f\left(x\right)\right\|}_{2}\leq K{\left\|\xi \right\|}_{2}.$$

Applying a first-order Taylor expansion to *f*(***x*** + ***ξ***) yields the gradient constraint:

(Equation 5) $$\frac{{\left\|\frac{\partial f\left(x\right)}{\partial x}\xi \right\|}_{2}}{{\left\|\xi \right\|}_{2}}\leq K.$$

Consequently, the Lipschitz constant *k*_0_ is mathematically bounded by the maximum singular value (or spectral norm) of the Jacobian matrix:

(Equation 6) $$k_{0}=\underset{\xi \neq 0}{\sup}\frac{{\left\|\frac{\partial f\left(x\right)}{\partial x}\xi \right\|}_{2}}{{\left\|\xi \right\|}_{2}}=\underset{{\left\|\xi \right\|}_{2}=1}{\max}{\left\|\frac{\partial f\left(x\right)}{\partial x}\xi \right\|}_{2}=\sigma_{\max}\left(\frac{\partial f\left(x\right)}{\partial x}\right).$$

To systematically evaluate the stability of the ATFF module, we analyze the derivative of its output ***x***_*tf*_ with respect to the input sequence ***x***_*t*_ (a 1 × *n* row vector). According to Equation 1, this derivative is formulated as:

(Equation 7) $${\left(\frac{\partial x_{tf}}{\partial x_{t}}\right)}_{\text{ATFF}}=\frac{\partial x_{t\text{\_}wsh}}{\partial x_{t}}+I,$$

where ***I*** denotes the *n* × *n* identity matrix. Because the operations are applied element-wise, the Jacobian matrix $\frac{\partial x_{t\text{\_}wsh}}{\partial x_{t}}$ is diagonal:

(Equation 8) $$\begin{array}{cc} \frac{\partial x_{t\text{\_}wsh}}{\partial x_{t}} & =\left(\begin{array}{cccc} \frac{\partial x_{t\text{\_}wsh_{0}}}{\partial x_{t_{0}}} & \frac{\partial x_{t\text{\_}wsh_{0}}}{\partial x_{t_{1}}} & \ldots & \frac{\partial x_{t\text{\_}wsh_{0}}}{\partial x_{t_{n-1}}}\\ \frac{\partial x_{t\text{\_}wsh_{1}}}{\partial x_{t_{0}}} & \frac{\partial x_{t\text{\_}wsh_{1}}}{\partial x_{t_{1}}} & \ldots & \frac{\partial x_{t\text{\_}wsh_{1}}}{\partial x_{t_{n-1}}}\\ \vdots & \vdots & \ddots & \vdots \\ \frac{\partial x_{t\text{\_}wsh_{n-1}}}{\partial x_{t_{0}}} & \frac{\partial x_{t\text{\_}wsh_{n-1}}}{\partial x_{t_{1}}} & \ldots & \frac{\partial x_{t\text{\_}wsh_{n-1}}}{\partial x_{t_{n-1}}} \end{array}\right)\\ & =diag\left(\frac{\partial x_{t\text{\_}wsh_{0}}}{\partial x_{t_{0}}},\frac{\partial x_{t\text{\_}wsh_{1}}}{\partial x_{t_{1}}},\ldots ,\frac{\partial x_{t\text{\_}wsh_{n-1}}}{\partial x_{t_{n-1}}}\right). \end{array}$$

Applying the chain and product rules, the non-zero diagonal elements evaluate to:

(Equation 9) $$\begin{array}{cc} \frac{\partial x_{t\text{\_}wsh_{i}}}{\partial x_{t_{i}}} & =\frac{\partial \left[sig\left(w_{t}x_{t_{i}}\right)w_{t}x_{t_{i}}\right]}{\partial x_{t_{i}}}\\ & =w_{t}sig\left(w_{t}x_{t_{i}}\right)+w_{t}^{2}x_{t_{i}}sig\left(w_{t}x_{t_{i}}\right)\left[1-sig\left(w_{t}x_{t_{i}}\right)\right],\;0\leq i\leq n-1. \end{array}$$

To analytically examine the properties of this derivative, we define a multiplier function *H*(*z*):

(Equation 10) $$H\left(z\right)=sig\left(z\right)+zsig\left(z\right)\left[1-sig\left(z\right)\right].$$

Substituting $z=w_{t}x_{t_{i}}$, Equation 9 simplifies to $w_{t}H\left(w_{t}x_{t_{i}}\right)$. Consequently, the overall diagonal elements of the ATFF Jacobian become:

(Equation 11) $${\left(\frac{\partial x_{tf_{i}}}{\partial x_{t_{i}}}\right)}_{\text{ATFF}}=w_{t}H\left(w_{t}x_{t_{i}}\right)+1.$$

In contrast, based on Equation 2, the WTFF module operates as a linear transformation. Its derivative with respect to ***x***_*t*_ forms a constant diagonal matrix:

(Equation 12) $${\left(\frac{\partial x_{tf}}{\partial x_{t}}\right)}_{\text{WTFF}}=\frac{\partial x_{t\text{\_}\alpha }}{\partial x_{t}}+I=\left(w_{\alpha }+1\right)I.$$

Based on the derived Jacobian matrices, we analytically formulate the theoretical Lipschitz constants for both modules. For the WTFF module, the uniform linear gradient governs its Lipschitz constant *K*_WTFF_. Thus, the mathematical bound is linearly dependent on the global scaling weight *w*_*α*_:

(Equation 13) $$K_{\text{WTFF}}=\max_{x_{t}}{\left\|{\left(\frac{\partial x_{tf}}{\partial x_{t}}\right)}_{\text{WTFF}}\right\|}_{2}=|1+w_{\alpha }|.$$

For the ATFF module, since its Jacobian matrix is diagonal, the Lipschitz constant *K*_ATFF_ corresponds to the supremum of the local derivatives across all data points $x_{t_{i}}$:

(Equation 14) $$K_{\text{ATFF}}=\max_{x_{t_{i}}}\left|1+w_{t}H\left(w_{t}x_{t_{i}}\right)\right|.$$

The dynamic gating mechanism $sig\left(w_{t}x_{t_{i}}\right)$ selectively modulates the input features. By substituting $z=w_{t}x_{t_{i}}$, the behavior of *H*(*z*) can be evaluated. When the product *z* is negative, which frequently corresponds to background noise, *H*(*z*) approaches zero. This attenuation neutralizes the influence of *w*_*t*_ and bounds the local derivative toward 1:

(Equation 15) $$\underset{z\to -\infty }{\lim}\left|1+w_{t}H\left(z\right)\right|=1.$$

Even when *z* is positive, the non-linear modulation mitigates uniform gradient accumulation. Numerical evaluations demonstrate that *H*(*z*) is bounded approximately within the interval [−0.100, 1.100]. This data-dependent limitation establishes a mathematical upper bound for the local Lipschitz constant:

(Equation 16) $$K_{\text{ATFF}}\leq \max\left(\left|1-0.100w_{t}|,|1+1.100w_{t}\right|\right)\approx 1+1.100|w_{t}|.$$

In practical signal processing scenarios, the normalized chaotic signals $x_{t_{i}}$ typically fluctuate within the interval [−1, 1]. From a purely mathematical perspective, *w*_*t*_ and *w*_*α*_ are unconstrained learnable parameters. Therefore, their final magnitudes are not determined by predetermined algebraic rules but rather governed by the optimization dynamics during network training.

For the WTFF module, the transformation is purely linear. To effectively separate the target chaotic characteristics and prevent them from being submerged in background noise, the optimization process inherently drives the network to learn a relatively large global scaling weight *w*_*α*_. As analytically shown in Equation 13, this substantial uniform amplification proportionally inflates the theoretical bound *K*_WTFF_.

In contrast, the ATFF module incorporates the non-linear, data-dependent gating function $sig\left(w_{t}x_{t_{i}}\right)$. This dynamic mechanism selectively emphasizes salient features while effectively attenuating negative noise components. Because this structural characteristic provides an independent capacity for feature distinguishability, it alleviates the necessity for the network to rely on a massively scaled global weight. Consequently, the gradient optimization typically yields an optimal weight magnitude |*w*_*t*_| that is smaller than |*w*_*α*_|.

By synthesizing these distinct optimization tendencies with the analytically derived limits from (Equation 13), (Equation 16), the mathematical bounds yield the following expected relationship upon training convergence:

(Equation 17) $$K_{\text{ATFF}}\leq 1+1.100|w_{t}|<|1+w_{\alpha }|=K_{\text{WTFF}}.$$

Rather than an unconditional absolute mathematical axiom, Equation 17 characterizes a systematic structural advantage. The combination of moderated learned weights (facilitated by the gating mechanism) and the intrinsic boundary of the multiplier function *H*(*z*) provides a reliable analytical explanation for the empirical observation that ATFF consistently maintains a smaller Lipschitz constant than WTFF.

#### Lipschitz constants in chaos identification experiments

To validate the theoretical bounds, we numerically evaluate the empirical Lipschitz constants of ATFF and WTFF across four structural configurations: ATFF-P8, WTFF-P8, ATFF-P2, and WTFF-P2. As summarized in Table 5, the ATFF-based structures consistently exhibit lower Lipschitz constants than their WTFF-based counterparts under various noise conditions. Notably, at noise levels of 15, 12.5, and 10 dB, the Lipschitz constants for ATFF-P8 are approximately half the magnitude of those for WTFF-P8.

**Table 5:** The Lipschitz constants obtained from simulation experiments

SNR (dB)	ATFF-P8	WTFF-P8	ATFF-P2	WTFF-P2
20	1.3811	2.4129	0.8699	1.7223
17.5	1.1656	2.3496	0.8808	1.7105
15	0.8849	1.9133	0.8500	1.1905
12.5	0.8621	2.0013	0.8797	1.4205
10	0.8595	1.6708	0.8676	1.2650

A smaller Lipschitz constant mathematically implies that a neural network system is more stable and less sensitive to input perturbations. These numerical observations align with the performance metrics presented in Tables 2 and 3, where ATFF-based structures demonstrate superior identification accuracy and stability. For instance, at a 17.5 dB SNR, the accuracy of WTFF-P2 decreases by 0.35% relative to non-WT-P2, indicating a vulnerability to noise. Conversely, ATFF-P2 maintains stable performance, yielding a 0.14% accuracy improvement over non-WT-P2. This divergence in robustness is analytically reflected in their respective Lipschitz constants: ATFF-P2 yields a value of 0.8808, which is considerably lower than the 1.7105 observed for WTFF-P2.

#### Internal relationship between Lipschitz constant and noise robustness

While the theoretical bounds and empirical numerical results establish that *K*_ATFF_ < *K*_WTFF_, it is essential to analytically connect, this mathematical property to the model’s robustness in chaotic signal identification tasks.

In practical scenarios, the clean chaotic signal ***x***_*t*_ is corrupted by background noise ***ξ***, resulting in the observed noisy input ${\tilde{x}}_{t}=x_{t}+\xi$. According to the definition of Lipschitz continuity in Equation 4, the deviation in the extracted features caused by the noise perturbation is mathematically bounded by:

(Equation 18) $${\left\|f\left({\tilde{x}}_{t}\right)-f\left(x_{t}\right)\right\|}_{2}\leq K{\left\|\xi \right\|}_{2}.$$

Equation 18 analytically indicates that the Lipschitz constant *K* serves as the maximum noise amplification factor of the neural network module. Chaotic signals are inherently characterized by complex, dense phase-space trajectories and broad continuous frequency spectra. To successfully identify a specific type of chaos, the network must reliably extract features that represent the underlying invariants of the chaotic system. These intrinsic deterministic invariants are highly susceptible to being masked if the external noise ***ξ*** is amplified during forward propagation.

When a feature extraction module exhibits a larger Lipschitz constant, such as the WTFF module (1.6708 ≤ *K*_WTFF_ ≤ 2.4129), the background noise vector ***ξ*** is amplified by a significantly larger factor. This amplified error distorts the delicate feature manifold, blurring the decision boundaries between different chaotic classes. Consequently, as the SNR decreases (e.g., from 20 to 10 dB), the feature representations become entangled, leading to a marked degradation in identification accuracy.

Conversely, the ATFF maintains a smaller Lipschitz constant (0.8595 ≤ *K*_ATFF_ ≤ 1.3811). This mathematical property ensures that the upper bound of the noise amplification, $K_{\text{ATFF}}{\left\|\xi \right\|}_{2}$, is effectively constrained. From a signal processing perspective, the smaller *K*_ATFF_ functions as an analytical stabilizer. By preventing high-frequency noise from overwhelming the intrinsic chaotic trajectory, the structural integrity of the chaotic features is preserved in the deep embedding space. This systematic connection provides a reliable theoretical explanation for why architectures equipped with ATFF consistently demonstrate superior identification performance and enhanced robustness against environmental interference.

### Superiority of non-WT-P8 over non-WT-P2 in chaos identification

A structure with fewer modules introduces less interference, thereby making the pooling contribution more evident. We primarily analyzed the two structures, non-WT-P8 and non-WT-P2, because they do not contain wavelet transform modules. We analyze the properties of the data generated by non-WT-P8 and non-WT-P2. The pooled feature sequences processed by non-WT-P8 and non-WT-P2 each contain 15 types of chaotic data. For non-WT-P8, the single-channel data length is 16,000 per chaos type, yielding a total of 240,000 samples across all 15 chaos types. For non-WT-P2, the single-channel data length is 64,000 per chaos type, totaling 960,000 samples. For both datasets, the pairwise *L*_2_ distances between all 15 chaotic types have been computed. The resulting average distances are presented in Table 6.

**Table 6:** The average pairwise *L*_2_ distance among the 15 chaotic types

SNR (dB)	Non-WT-P8	Non-WT-P2
20	1.289 × 10^5^	1.100 × 10^5^
17.5	1.182 × 10^5^	9.110 × 10^4^
15	8.698 × 10^4^	6.648 × 10^4^
12.5	6.087 × 10^4^	5.535 × 10^4^
10	2.826 × 10^4^	2.343 × 10^4^

According to Table 6, the *L*_2_ distances for the non-WT-P8 are consistently greater than those for the non-WT-P2 across all five SNRs. In the last column of Table 3, the non-WT-P8 exhibits overall higher chaos identification accuracy compared to the non-WT-P2. A larger *L*_2_ distance between different types of chaotic data generally indicates greater discriminability among the data, which, in turn, leads to improved identification accuracy. Thus, the findings from Table 6 are consistent with those from Table 3. Table 6 shows that *L*_2_ distances for both non-WT-P8 and non-WT-P2 decrease as SNR drops. In Table 2, the identification accuracy of non-WT-P8 for chaotic data declines with reduced SNR. Non-WT-P2 shows a similar trend. A lower SNR means noise dominates the data more, reducing the distinctiveness between types (as shown by smaller *L*_2_ distances) and harming identification accuracy. Thus, the SNR and *L*_2_ distance relationship in Table 6 matches the SNR-accuracy pattern in Table 2.

As shown in Table 3, the identification accuracy of non-WT-P8 exceeds that of non-WT-P2 by 0.96% at 10 dB. We therefore analyze the dynamic time warping (DTW) distances at this specific SNR to explore the underlying reason.^13^ For two time series ***x*** = (*x*_1_, *x*_2_, ⋯ , *x*_*n*_) and ***y*** = (*y*_1_, *y*_2_, ⋯ , *y*_*m*_), the calculation method of DTW is introduced as follows.

(1) Let $d\left(x_{i_{k}},y_{j_{k}}\right)$ denote the distance between two points, i.e., $x_{i_{k}}$ and $y_{j_{k}}$, where *i*_*k*_ ranges from 1 to *n*, *j*_*k*_ ranges from 1 to *m*, and *k* ranges from 1 to *T*.
(2) Extract 2*T* data points from the sequences ***x*** and ***y*** and compute the corresponding distances. That is, a cumulative distance needs to be calculated $d\left(x,y\right)=\overset{T}{\underset{k=1}{\sum }}d\left(x_{i_{k}},y_{j_{k}}\right)$.
(3) Identify the optimal *i*_*k*_ and *j*_*k*_ that minimize the distance between sequences ***x*** and ***y***. That is, compute

(Equation 19) $$DTW\left(x,y\right)=\underset{i_{k},j_{k}}{\min}d\left(x,y\right)=\underset{i_{k},j_{k}}{\min}\overset{T}{\underset{k=1}{\sum }}d\left(x_{i_{k}},y_{j_{k}}\right).$$

At 10 dB, the average DTW distances are 4.712 × 10^5^ for non-WT-P8 and 4.439 × 10^5^ for non-WT-P2. The non-WT-P8 value is 6.15% higher. The average L_2_ distances are 2.826 × 10^4^ for non-WT-P8 and 2.343 × 10^4^ for non-WT-P2. This shows a 20.61% increase for non-WT-P8. Both DTW and *L*_2_ distances are greater for non-WT-P8 than for non-WT-P2 at 10 dB.

### Comparison of SFFT-CC and CDC

This section compares two methods for extracting features from causal chaotic sequences: the SFFT-CC used in this work and the classical causal dilated convolution (CDC). First, we analyze the number of multiplications needed for SFFT-CC. Let *N*_*ch*_ be the number of channels for both input and output data, and *n* the length of the input and output sequences. Here, *N*_*ch*_ = *αn*, where 0 < *α* < + *∞*. SFFT-CC requires *N*_mult_s_ = *N*_*ch*_*n* log_2_*n* multiplications.^14^^,^^15^ CDC needs $N_{mult\text{\_}c}={\left(N_{ch}\right)}^{2}k_{\mathrm{size}}n$ multiplications, where *k*_*size*_ is the kernel size.^16^^,^^17^ The ratio of CDC to SFFT-CC multiplications is therefore

(Equation 20) $$\frac{N_{mult\text{\_}c}}{N_{mult\text{\_}s}}=\frac{{\left(N_{ch}\right)}^{2}k_{\mathrm{size}}n}{N_{ch}n\log_{2}n}=\alpha k_{\mathrm{size}}\frac{n}{\log_{2}n}=\alpha k_{\mathrm{size}}f\left(n\right).$$

The derivative of $f\left(n\right)=\frac{n}{\log_{2}n}$ is

(Equation 21) $$\frac{\partial f\left(n\right)}{\partial n}=\frac{\log_{2}n-\frac{1}{\ln2}}{{\left(\log_{2}n\right)}^{2}}=\frac{\log_{2}n-\log_{2}e}{{\left(\log_{2}n\right)}^{2}}=\frac{\log_{2}\left(\frac{n}{e}\right)}{{\left(\log_{2}n\right)}^{2}},$$

where the sequence length *n* is greater than *e*. The derivative of *f*(*n*) is always greater than 0, so *f*(*n*) is monotonically increasing within its domain. With *α* = 1, *k*_*size*_ = 2 and *n* = 32, the ratio given by Equation 20 is $\frac{2\times 32}{5}=12.8$. In this scenario, CDC requires 12.8 times as many multiplications as SFFT-CC. To ensure a fair comparison, we set both the input and output channels of the CDC to $\frac{n}{2}$, which resulted in $N_{mult\text{\_}c}={\left(\frac{N_{ch}}{2}\right)}^{2}k_{\mathrm{size}}n=\frac{{\left(N_{ch}\right)}^{2}}{4}k_{\mathrm{size}}n$. Rewrite Equation 20 as

(Equation 22) $$\frac{N_{mult\text{\_}c}}{N_{mult\text{\_}s}}=\frac{{\left(N_{ch}\right)}^{2}k_{\mathrm{size}}n}{4N_{ch}n\log_{2}n}=\alpha k_{\mathrm{size}}\frac{n}{4\log_{2}n}.$$

With *α* = 1, *k*_*size*_ = 2 and *n* = 32, the ratio given by Equation 22 is $\frac{2\times 32}{4\times 5}=3.2$. Reducing the multiplication ratio from 12.8 to 3.2 yields a far more equitable baseline.

We present a theoretical mathematical analysis of how SFFT-CC and CDC compute. In SFFT-CC, the input is *X* = {*x*_0_, *x*_1_, …, *x*_*n*−1_} and the weights are *W* = {*w*_0_, *w*_1_, …, *w*_*n*−1_}. We compute the causal cross-correlation between *X* and *W*, then apply the activation function *f*_*σ*_(⋅). The explicit expansion is:

(Equation 23) $$\begin{array}{cc} & y_{0}=f_{\sigma }\left(w_{0}x_{0}\right)\\ & y_{1}=f_{\sigma }\left(w_{0}x_{1}+w_{1}x_{0}\right)\\ & y_{2}=f_{\sigma }\left(w_{0}x_{2}+w_{1}x_{1}+w_{2}x_{0}\right)\\ & \;\;\;\vdots \\ & y_{i}=f_{\sigma }\left(\overset{i}{\underset{k=0}{\sum }}w_{k}x_{i-k}\right),\;0\leq i\leq n-1. \end{array}$$

**Figure 4:**
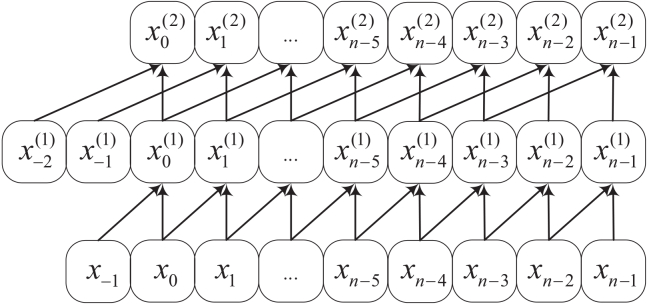
The structure of causal dilated convolution

To keep multiplication counts comparable to SFFT-CC, we set *k*_*size*_ to 2 and limit CDC to two layers. As shown in Figure 4, CDC uses three sequences: the input *X*, the first layer’s intermediate result $X^{\left(1\right)}=\left\{x_{0}^{\left(1\right)},x_{1}^{\left(1\right)},\ldots ,x_{n-1}^{\left(1\right)}\right\}$, and the second layer’s final result *X*^(2)^, where the superscripts in parentheses denote the network layer indices.

**Figure 5:**
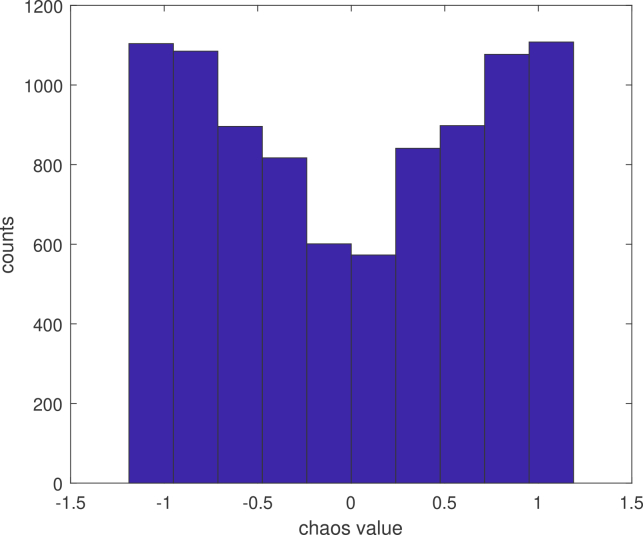
Histogram of Tanh-Gauss map

**Figure 6:**
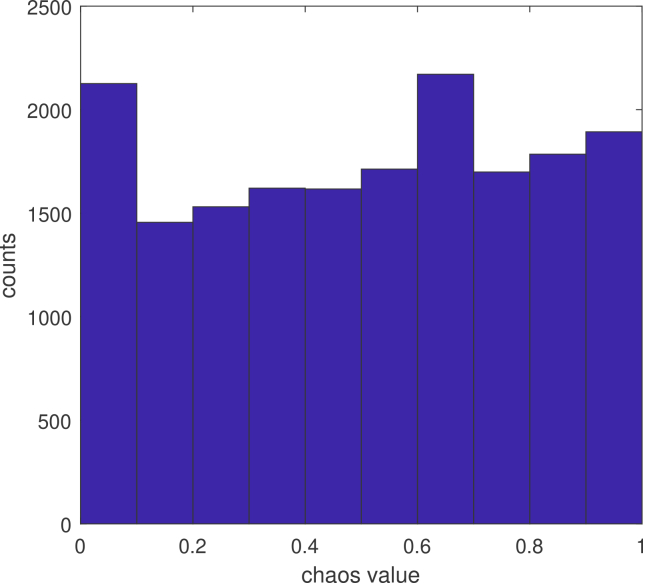
Histogram of Tanh-exp map

**Figure 7:**
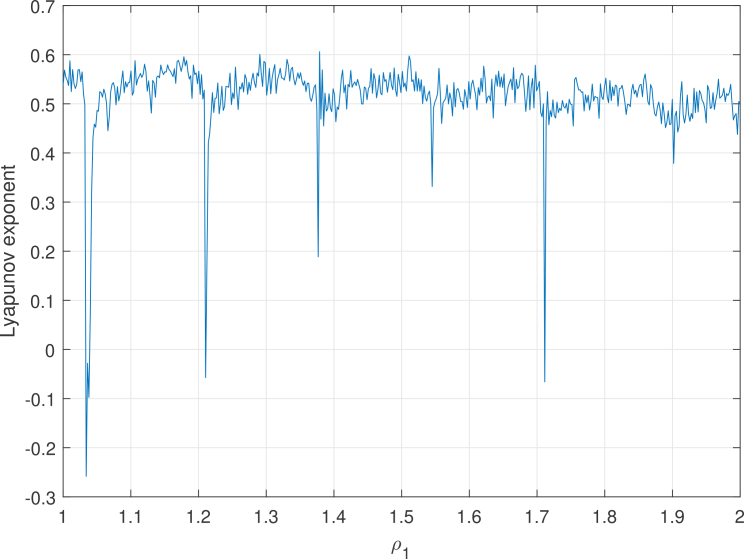
The Lyapunov exponent of Tanh-exp map

**Figure 8:**
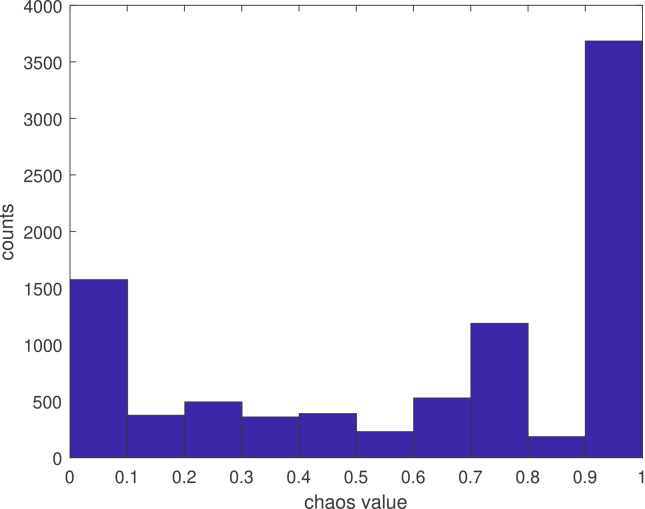
The Lyapunov exponent of bi-sigmoid map

**Figure 9:**
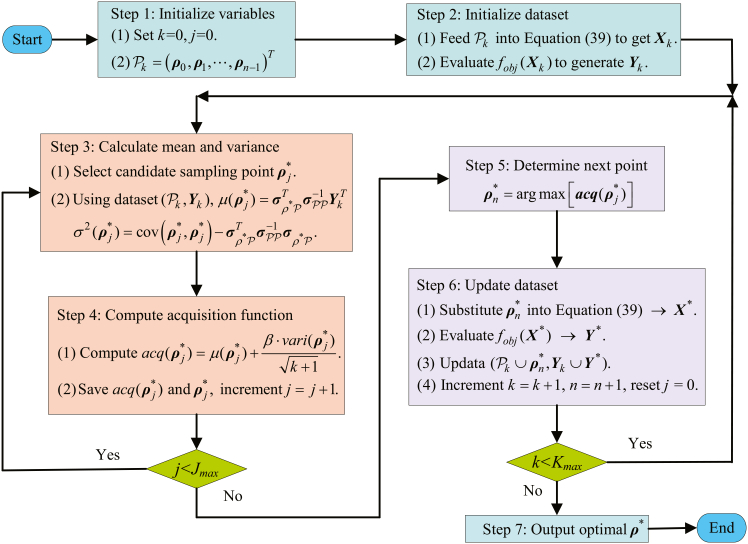
The flow chart of Bayesian optimization

**Figure 10:**
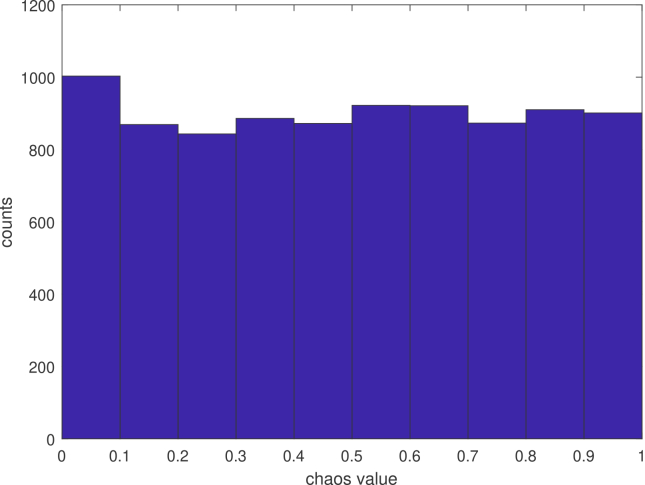
Histogram of Swish-modulo map

**Figure 11:**
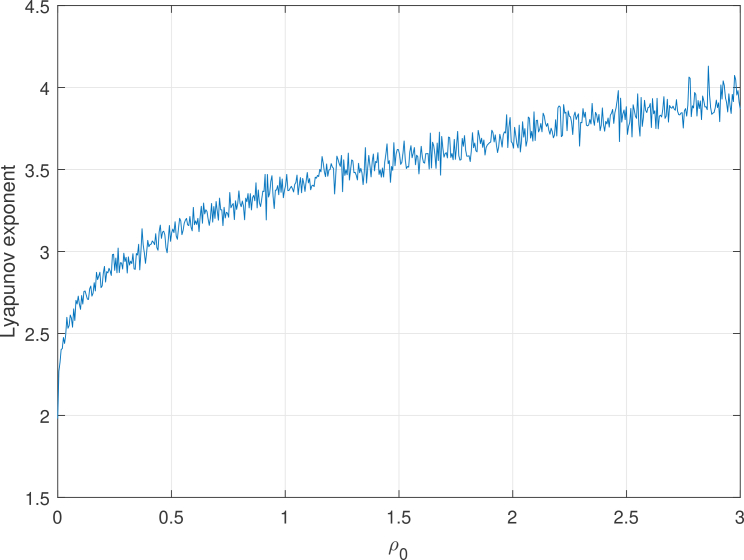
Lyapunov exponent of Swish-modulo map

**Figure 12:**
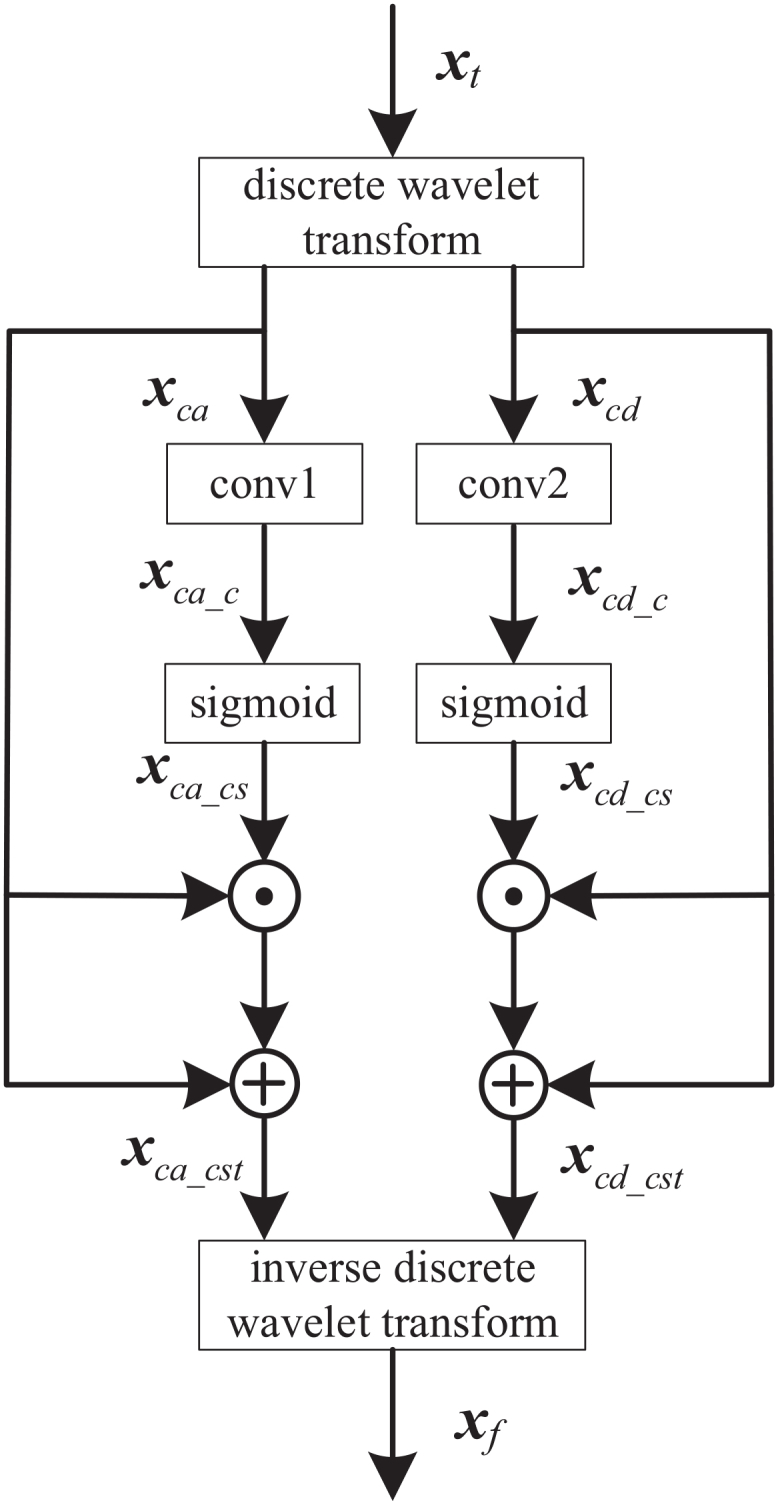
The structure of the wavelet module

**Figure 13:**
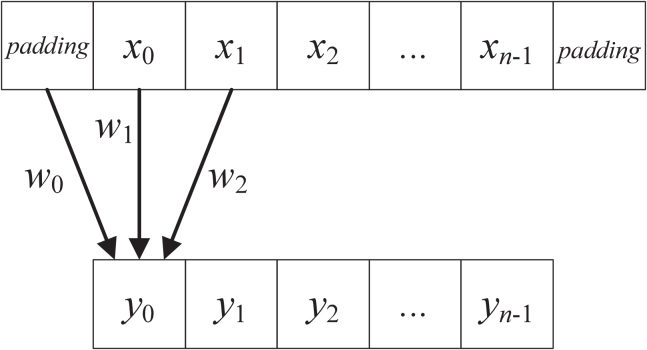
Conventional convolution computation

**Figure 14:**
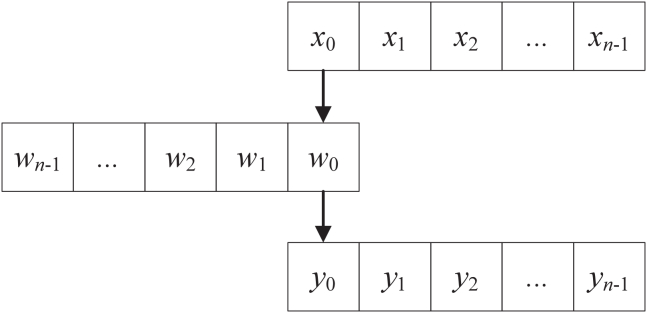
Causal alignment operation 1

**Figure 15:**
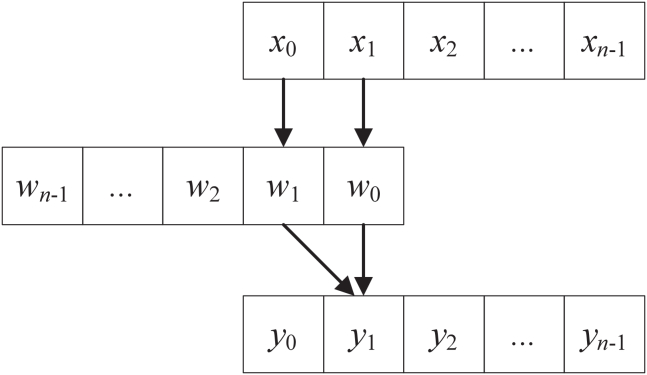
Causal alignment operation 2

The functional relationship mapping the sequence *X* to *X*^(1)^ (with a dilation rate of 1) is rigorously expressed as:

(Equation 24) $$\begin{array}{cc} & x_{n-1}^{\left(1\right)}=f_{\sigma }\left(w_{0}^{\left(0\right)}x_{n-1}+w_{1}^{\left(0\right)}x_{n-2}\right)\\ & x_{n-2}^{\left(1\right)}=f_{\sigma }\left(w_{0}^{\left(0\right)}x_{n-2}+w_{1}^{\left(0\right)}x_{n-3}\right)\\ & \;\;\;\vdots \\ & x_{i}^{\left(1\right)}=f_{\sigma }\left(\overset{1}{\underset{k=0}{\sum }}w_{k}^{\left(0\right)}x_{i-k}\right),\;\text{with}\;x_{-1}=0,\;0\leq i\leq n-1. \end{array}$$

The relationship mapping the intermediate sequence *X*^(1)^ to the final output *X*^(2)^ (with a dilation rate of 2) is given by:

(Equation 25) $$\begin{array}{cc} x_{n-1}^{\left(2\right)} & =f_{\sigma }\left(w_{0}^{\left(1\right)}x_{n-1}^{\left(1\right)}+w_{1}^{\left(1\right)}x_{n-3}^{\left(1\right)}\right)\\ & =f_{\sigma }\left[w_{0}^{\left(1\right)}f_{\sigma }\left(w_{0}^{\left(0\right)}x_{n-1}+w_{1}^{\left(0\right)}x_{n-2}\right)+w_{1}^{\left(1\right)}f_{\sigma }\left(w_{0}^{\left(0\right)}x_{n-3}+w_{1}^{\left(0\right)}x_{n-4}\right)\right]\\ & \;\;\;\vdots \\ x_{i}^{\left(2\right)} & =f_{\sigma }\left(\overset{1}{\underset{k=0}{\sum }}w_{k}^{\left(1\right)}x_{i-2k}^{\left(1\right)}\right)=f_{\sigma }\left(\overset{1}{\underset{k=0}{\sum }}w_{k}^{\left(1\right)}f_{\sigma }\left(\overset{1}{\underset{m=0}{\sum }}w_{m}^{\left(0\right)}x_{i-2k-m}\right)\right),\\ & \text{with}\;x_{-1}^{\left(1\right)}=x_{-2}^{\left(1\right)}=0,\;0\leq i\leq n-1. \end{array}$$

Within the CDC framework, the computation for each element in the output sequence *X*^(2)^ involves only a local receptive field of four elements from the input sequence *X*. This constrained connectivity may be insufficient to comprehensively capture the characteristic information in the input sequence. Conversely, each output of the SFFT-CC is associated with all previous inputs. In particular, the final output term, *y*_*n*−1_, is linked to every input element *x*_*i*_, since $y_{n-1}=f_{\sigma }\left(\overset{n-1}{\underset{k=0}{\sum }}w_{k}x_{n-k-1}\right)$. The functional mapping between the input and output in the SFFT-CC is structurally analogous to that expressed in the universal approximation theorem.^18^^,^^19^ This analogous form underpins SFFT-CC’s ability to effectively capture the characteristics of the chaotic sequence.

A comparative experiment between SFFT-CC and CDC was conducted at five different SNRs according to Equation 22, with the results summarized in Table 7.

**Table 7:** Performance comparison of SFFT-CC and CDC

		SFFT-CC	CDC	SFFT-CC vs. CDC
Accuracy	20 dB	95.64%	94.00%	1.64%
	17.5 dB	93.53%	88.11%	5.42%
	15 dB	88.50%	83.69%	4.81%
	12.5 dB	82.83%	78.26%	4.57%
	10 dB	75.01%	71.71%	3.30%
Time per 100 epochs		216.26s	9239.98s	1:43
Number of multiplications		*n*^2^ log_2_*n*	$\frac{n^{3}}{2}$	$\frac{2\log_{2}n}{n}$

For a fair comparison between the two methods, identical training parameters and computational hardware environments are adopted. To ensure the exact reproducibility of the execution time benchmark, all experiments were executed on a laptop computer equipped with an NVIDIA GeForce MX150 GPU (2 GB), an Intel Core i5-8250U CPU, and 24 GB of physical RAM, running on the Windows operating system with PyTorch and CUDA. The training process is conducted over 100 epochs using a batch size of 128. The Adam optimizer is employed with a learning rate of 0.005 for gradient optimization, while cross-entropy loss is utilized as the loss function.

Across the five SNR conditions tested, the SFFT-CC outperforms CDC in accuracy for chaotic signal identification. This performance advantage is most pronounced at 17.5 dB, where the SFFT-CC achieves a 5.3% higher accuracy than the CDC. Furthermore, we employ the standard torch.cuda.Event function to benchmark the execution time over 100 training epochs for both SFFT-CC and CDC. The CDC scheme consumed approximately 9,239.98 s (2.567 h), whereas the SFFT-CC algorithm required only 216.26 s (3.604 min) under identical testing conditions. This result indicates that the training time of CDC was 43 times that of SFFT-CC.

We attribute the slow CDC execution to its memory access pattern. The dilated operations within CDC require it to skip adjacent elements and access memory in a non-contiguous, jumping pattern. This cache-unfriendly behavior significantly reduces cache hit rates, as the required data are often not present in the cache. Consequently, the GPU is forced to fetch data from the much slower global memory more frequently, which substantially increases the actual computation time. As specified by Equation 22 for the case where *α* = 1 and *k*_*size*_ = 2, we present the number of multiplications consumed by both SFFT-CC and CDC in Table 7. In general, the CDC consumes more multiplications than the SFFT-CC.

### Comparison with existing approaches

To comprehensively highlight the practical engineering advantages of the proposed non-WT-P8 structure, we compare it with existing chaos identification methods across multiple dimensions. Because absolute execution time and dynamic memory consumption heavily depend on specific hardware platforms, we adopt the magnitude of trainable parameters as an objective proxy for the memory footprint, and the exact number of multiplications as a rigorous, hardware-independent metric for real-time computational efficiency. Let *n* denote the input sequence length, *k* the convolution kernel size, and *N*_*ch*_ the number of channels (assuming equal input and output channels for simplicity). The comparative results are summarized in Table 8.

**Table 8:** Comparison of chaos identification methods employed in different studies

method	Chaos type	Identification accuracy	Magnitude of parameters	Number of multiplications
ResNet^2^	logistic	100%	$∼1\times 10^{6}$	$∼nkN_{ch}^{2}$
ResNet^2^	cubic	97%	$∼1\times 10^{6}$	$∼nkN_{ch}^{2}$
MCDCNN^2^	logistic	98%	$∼1\times 10^{4}$	$∼nkN_{ch}^{2}$
MCDCNN^2^	cubic	88%	$∼1\times 10^{4}$	$∼nkN_{ch}^{2}$
LKCNN^5^	logistic	98.8%	$∼1\times 10^{4}$	$∼nkN_{ch}^{2}$
LKCNN^5^	circle	89.8%	$∼1\times 10^{4}$	$∼nkN_{ch}^{2}$
CNN^4^	logistic	90%	$∼1\times 10^{4}$	$∼nkN_{ch}^{2}$
Multimodal DNN^9^	simulated and real-world	> 96%	> 1 × 10^6^	$∼n^{2}k^{2}N_{ch}^{2}$
Non-WT-P8 (ours)	15 types of chaos	99.73%	2 × 10^3^	*N*_*ch*_*n* log_2_*n*

**Table 9:** Comprehensive nomenclature of abbreviations and principal mathematical symbols

Abbreviation/Symbol	Description
**Six proposed network variants (ablation structures)**	

ATFF-P8/ATFF-P2	architecture incorporating the wavelet module and attention-based time-frequency feature fusion, combined with 1 × 8/1 × 2 average pooling.
WTFF-P8/WTFF-P2	architecture incorporating the wavelet module and weighted time-frequency feature fusion, combined with 1 × 8/1 × 2 average pooling.
Non-WT-P8/non-WT-P2	simplified architecture explicitly excluding both the wavelet transform and fusion modules, utilizing directly 1 × 8/1 × 2 average pooling.

**Other architectures and core modules**	

ATFF/WTFF	attention-based/weighted time-frequency feature fusion
SFFT-CC	spectral-inversion-free fast fourier transform-based cross-correlation
CDC	causal dilated convolution
AvgP-8/AvgP-2	1 × 8 and 1 × 2 average pooling operations
ResNet/MCDCNN	residual neural network/multi-channel deep convolutional neural network
LKCNN	large kernel convolutional neural network

**Algorithms, metrics, and chaotic maps**	

DTW	dynamic time warping (utilized for time series distance measurement)
SNR	signal-to-noise ratio (measured in dB to simulate background noise)
PWLCM	piecewise linear chaotic map (map 9 in the evaluated chaos models)
ECG	electrocardiogram (real-world biomedical dataset for generalizability testing)

**Data, features, and optimization parameters**	

$x_{t},x_{t_{i}}$	the 1D raw chaotic sequence and its specific data point at step *i*
***x***_*f*_, ***x***_*tf*_, ***x***_*tfc*_	frequency-domain features, fused time-frequency features, and refined features
*N*_*ch*_, *k*_*size*_	number of network channels, receptive field kernel size
*ρ*, *ρ*_0_, *ρ*_1_ …	bifurcation control parameters for specific chaotic maps
$\rho_{j}^{*},f_{\mathrm{obj}}\left(\cdot \right)$	candidate parameter sampling point and objective function in Bayesian optimization

**Network weights and mathematical functions**	

*K*, *k*_0_	Lipschitz constants mathematically determining system input-output stability
*w*_*t*_, *w*_*f*_, *w*_*α*_, *w*_*β*_	learnable fusion scaling weights in the ATFF and WTFF modules
*W*, *w*_0_, *w*_*k*_, …	learnable weight parameters utilized in neural network layers
*N*_mult_s_, *N*_mult_c_	exact number of multiplication operations required for SFFT-CC and CDC
*ω*	angular frequency utilized in the discrete wavelet transform
*f*_*σ*_(⋅), sig(⋅)	standard non-linear sigmoid activation function

Using ResNet, logistic identification achieves 100% accuracy but requires a large memory footprint of about 1 × 10^6^ parameters.^2^ The lightweight MCDCNN network attains 98% accuracy with around 1 × 10^4^ parameters.^2^ LKCNN yields 98.8% accuracy for logistic identification, while circle-type chaos identification reaches 89.8%.^5^ The standard CNN structure delivers 90% accuracy.^4^ Because these traditional networks rely on classical 1D spatial convolutions, their multiplication operations scale quadratically with the channel size $\left(N_{ch}^{2}\right)$.

Recently, Giuseppi et al.^9^ proposed a multimodal deep neural network combining information from 1D time series, 2D recurrence plots, and spectrograms. While transforming 1D chaotic sequences into *n* × *n* 2D images provides a comprehensive representation, it exacerbates the computational burden. The processing requires 2D convolutions, which drives the multiplication count up to approximately $n^{2}k^{2}N_{ch}^{2}$ and increases the parameter scale to exceed 1 × 10^6^. Such substantial memory and computational overhead may limit its real-time streaming capability.

In contrast, our proposed non-WT-P8 structure directly processes 1D raw sequences, employing only 2 × 10^3^ parameters and achieving 99.73% accuracy across 15 types of chaos. This accuracy is slightly lower than the 100% reported in the literature^2^ for single logistic identification, by 0.27 percentage points. However, the parameter count of our adopted network structure decreases by two to three orders of magnitude.

More importantly, our method fundamentally optimizes the computational architecture. By leveraging the spectral-inversion-free FFT, the proposed causal cross-correlation captures temporal features globally across the entire sequence. Through frequency-domain processing, the quadratic channel dependency $\left(N_{ch}^{2}\right)$ inherent in traditional spatial CNNs is ingeniously eliminated, compressing the multiplication count to *N*_*ch*_*n* log_2_*n*. In typical deep learning scenarios, where *N*_*ch*_ is relatively large, avoiding the $N_{ch}^{2}$ term translates to a faster execution speed. Furthermore, the computations of deep learning models developed by other researchers typically utilize future inputs to generate current outputs, disregarding the causal characteristics of chaotic sequences. Our proposed non-WT-P8 explicitly maintains physical causality. Consequently, the proposed method demonstrates practical engineering advantages: it ensures a minimal physical memory footprint, respects temporal causality, and achieves high computational efficiency.

To summarize, this article investigates 15 distinct chaotic equations. We propose a neural network for chaos identification under different SNRs, including two key components: a CNN-based learnable wavelet module and an SFFT-based causal cross-correlation module. (1) We assess chaos identification under noise at five SNRs: 20, 17.5, 15, 12.5, and 10 dB. To test the robustness of our identification structure, we alter the initial values of 15 chaotic sequences at each SNR. (2) We introduce two fusion structures for the wavelet module: ATFF and WTFF. At 10 dB, ATFF-P8 improves accuracy by 1.42% compared to non-WT-P8, and ATFF-P2 outperforms non-WT-P2 by 1.06%. Furthermore, under certain conditions, we derive upper bounds on the Lipschitz constants for ATFF and WTFF. Both theory and experiments indicate that ATFF has a smaller Lipschitz constant than WTFF. Consequently, under various noise conditions, ATFF consistently improves accuracy and robustness, while WTFF is less robust. (3) We conduct ablation experiments. At 10 dB, non-WT-P8 uses 71.43% fewer parameters and achieves a 0.96% identification accuracy improvement over non-WT-P2. Furthermore, we conduct a theoretical analysis by comparing the *L*_2_ distances of the two configurations. The analysis results indicate that non-WT-P8 consistently has larger *L*_2_ distances than non-WT-P2 for all SNRs. Specifically, at 10 dB, the *L*_2_ distance of non-WT-P8 is 20.61% higher than that of non-WT-P2. The distances between different chaotic types in the non-WT-P8 dataset are greater, making the classes easier to distinguish. Consequently, this accounts for the higher identification accuracy of non-WT-P8 compared to non-WT-P2. (4) We present a detailed comparison of two methods for causal feature extraction: the SFFT-CC employed in this work and the CDC approach. The SFFT-CC offers several significant advantages over the CDC. SFFT-CC exhibits superior robustness across various noise levels, adheres to the form of the universal approximation theorem, and requires fewer multiplicative operations, resulting in faster execution times. (5) In the context of chaos identification, extracting dynamic features while maintaining physical sequence causality and computational efficiency is an essential consideration. Classical recurrent architectures, such as long short-term memory networks, naturally preserve temporal causality; however, their sequential execution mechanisms can limit hardware parallelization. Alternatively, some recent deep learning models transform 1D sequences into 2D spatial representations, which may alter the forward-evolving physical causality of the original signals. To balance these aspects, we employ the non-WT-P8 structure for causal computation. It preserves sequence causality while utilizing the SFFT-CC to reduce the multiplication operations to *N*_*ch*_*n* log_2_*n*. Operating with a lightweight footprint of approximately 2 × 10^3^ parameters, it achieves an accuracy of 99.73% under noise-free conditions. This result is comparable to those reported in the literature. In conclusion, the proposed chaos identification framework demonstrates certain advantages.

### Limitations of the study

While the proposed causal cross-correlation neural network offers potential advantages in multi-chaotic signal identification and computational efficiency, certain limitations should be acknowledged.

First, the current study primarily focuses on 15 classical chaotic maps and specific real-world ECG datasets. Although these maps cover a wide range of dynamic behaviors and constitute a substantially broader benchmark than that adopted in most existing chaos identification studies, the framework could be further enriched by incorporating additional chaotic systems (for example, the Lorenz, Rössler, and Chua systems). The generalizability of the network to higher dimensional hyperchaotic systems or to extremely non-stationary industrial signals remains to be further explored in future work. Second, the noise robustness analysis is performed under five representative additive Gaussian white noise levels, namely 20, 17.5, 15, 12.5, and 10 dB. While these settings encompass commonly encountered noise conditions, a finer-grained sweep over a denser set of SNRs would offer a more comprehensive characterization of the model’s resilience.

Finally, the current architecture is optimized for 1D time-series analysis; extending this causal framework to 2D spatiotemporal chaotic patterns represents a promising direction for subsequent research. We believe that addressing these aspects in future work will further strengthen and broaden the applicability of the chaos identification framework presented in this study.

## Resource availability

### Lead contact

Requests for further information and resources should be directed to and will be fulfilled by the lead contact, Chengbin Chen (ccb@fzu.edu.cn).

### Materials availability

This study did not generate new unique reagents.

### Data and code availability

• All data analyzed in this study have been deposited at GitHub and are publicly available as of the date of publication. The accession URL is listed in the key resources table: https://github.com/auevywtrsq/Multi-Chaotic-Signal-Identification.
• This paper does not report original code.
• Any additional information required to reanalyze the data reported in this paper is available from the lead contact upon request.

## Acknowledgments

This work is supported by Fuzhou Industry-Department Project under Grant 2025-ZD-020, Fujian Province Fund under Grant 2024H6013, Philosophy and Social Sciences Research Planning Program of Henan Province (no. 2024ZZX024), Scientific Research Project on the Development of Educational Informatization (no. 2025KT01005), Henan Postgraduate Education Reform and Quality Improvement Project (no. YJS2026YBGZZ46), Research Initiation Funding of Fuzhou University (no. XRC-26024), and Major Key Project of Pengcheng Laboratory (no. PCL2025A17-4). The Corresponding Authors (Chengbin Chen, Ente Guo).

## Author contributions

Conceptualization, methodology, and writing – original draft, B.W. and X.P.; investigation, X.P., C.C., and X.Z.; validation, B.W., X.P., C.C., and E.G.; data curation, B.W., X.P., and C.C.; writing – review, editing and funding acquisition, B.W., C.C., E.G., and X.Z.

## Declaration of interests

The authors declare no competing interests.

## STAR★Methods

### Key resources table

**Table:** 

REAGENT or RESOURCE	SOURCE	IDENTIFIER
**Deposited data**		

Raw and analyzed data	This paper	https://github.com/auevywtrsq/Multi-Chaotic-Signal-Identification

**Software and algorithms**		

Torch(version 1.10.1)	Open Source	https://pytorch.org
Torchvision(version 0.11.2)	Open Source	https://pytorch.org
Python(version 3.8.20)	Python Software Foundation	https://www.python.org
Numpy(version 1.24.3)	Python package	https://numpy.org
CUDA(version 10.2.89)	Proprietary	https://developer.nvidia.com/cuda-toolkit

### Experimental model and study participant details

Omitted as our study does not involve biological models. The influence of sex, gender, ancestry, race, and ethnicity is therefore not applicable to this work.

### Method details

#### Nomenclature and abbreviations

To enhance readability and clarify the mathematical expressions utilized across different modules and ablation studies, Table 9 provides a comprehensive summary of the principal mathematical symbols and common abbreviations employed in this paper, explicitly detailing the specific designations for the six structural variants evaluated in our ablation study.

#### Data generation based on 15 chaotic maps

Map 1: The Bernoulli shift map is defined by^20^

(Equation 26) $$x_{i+1}=\left\{\begin{array}{c} \rho_{0}x_{i}-\rho_{1},x_{i}>0\\ \rho_{0}x_{i}+\rho_{1},x_{i}\leq 0. \end{array}\right)$$

The Bernoulli shift map exhibits non-periodic dynamics as *ρ* approaches 2.

Map 2: The Chebyshev chaotic map is^21^

(Equation 27) $$x_{i+1}=\cos\left(\rho \arccos x_{i}\right).$$

The Chebyshev chaotic map demonstrates chaotic behavior when *ρ* ≥ 2.

Map 3: The circle map is defined as^22^

(Equation 28) $$x_{i+1}=\;\mathrm{mod}\;\left(x_{i}+\rho_{0}+\frac{\rho_{1}}{2\pi }\sin\left(2\pi x_{i}\right),1\right),$$

where ’mod’ denotes the modulo function. In the circle map, *ρ*_0_ is generally within [0,1). With *ρ*_0_ = 0, chaos arises when the absolute value of *ρ*_1_ exceeds 4.

Map 4: The expression for the cubic map is^23^

(Equation 29) $$x_{i+1}=\rho x_{i}\left(1-x_{i}^{2}\right).$$

The cubic map generates chaos for *ρ* ≥ 2.595.

Map 5: The Fuch map is described as^24^

(Equation 30) $$x_{i+1}=\cos\left(\frac{\rho }{x_{i}^{2}}\right),$$

The Fuch map exhibits chaotic behavior when 0 < *ρ* ≤ 1. The Fuch map is infinite and foldable under these conditions.

Map 6: The Gauss map is^25^

(Equation 31) $$x_{i+1}=e^{-\rho_{0}x_{i}^{2}}+\rho_{1},$$

The Gauss map is chaotic when *ρ*_0_ > 4.5 and *ρ*_1_ ∈ (−1, 1). The nonlinear Gauss map mimics a Gaussian probability density.

Map 7: The iterative map is written by^26^

(Equation 32) $$x_{i+1}=\sin\left(\frac{\rho \pi }{x_{i}}\right),$$

where *ρ* typically takes values within the interval 0 < *ρ* < 1.

Map 8: The logistic map is referred to as^27^^,^^28^

(Equation 33) $$x_{i+1}=\rho x_{i}\left(1-x_{i}\right).$$

The logistic map exhibits chaotic behavior as *ρ* increases from 0 to 4, and is fully chaotic at *ρ* = 4.

Map 9: The piecewise linear chaotic map (PWLCM) is described as follows^29^

(Equation 34) $$x_{i+1}=\left\{\begin{array}{c} f\left(x_{i},\rho \right)=\left\{\begin{array}{c} \frac{x_{i}}{\rho },0\leq x_{i}<\rho ,\\ \frac{x_{i}-\rho }{0.5-\rho },\rho \leq x_{i}<0.5, \end{array}\right)\\ f\left(1-x_{i},\rho \right),0.5\leq x_{i}<1, \end{array}\right)$$

where the *ρ* is a piecewise control factor with 0 < *ρ* < 0.5.

Map 10: The sine map is expressed in the mathematical form^30^

(Equation 35) $$x_{i+1}=\rho \sin\left(\pi x_{i}\right).$$

If the parameter *ρ* is chosen appropriately, the sine map tends to exhibit periodicity. Chaos occurs when *ρ* lies in the intervals (0.87, 0.93) and (0.95, 1).

Map 11: The Singer map is^31^

(Equation 36) $$x_{i+1}=\rho \left(7.86x_{i}-23.31x_{i}^{2}+28.75x_{i}^{3}-13.3x_{i}^{4}\right).$$

The Singer map exhibits chaotic behavior when *ρ* is within the interval [0.9, 1.08].

Map 12: The sinusoidal map is represented as^32^

(Equation 37) $$x_{i+1}=\rho x_{i}^{2}\sin\left(\pi x_{i}\right).$$

The sinusoidal map, as a sine-based variant, exhibits chaos under suitable parameter values.

Map 13: The tent map is^33^

(Equation 38) $$x_{i+1}=\left\{\begin{array}{c} \rho x_{i},x_{i}<0.5\\ \rho \left(1-x_{i}\right),x_{i}\geq 0.5. \end{array}\right)$$

The tent map requires careful parameter selection, as chaotic values seldom cover 0 to 1.^34^ Using an asymmetric tent map provides a more uniform chaotic distribution.

(Equation 39) $$x_{i+1}=\left\{\begin{array}{cc} \rho_{0}x_{i}, & x_{i}<\rho \\ \rho_{1}\left(1-x_{i}\right), & x_{i}\geq \rho . \end{array}\right)$$

We test the improved tent map using the Discrete Fourier Transform (DFT) algorithm.^35^ The parameter selection for this mapping is flexible. In Equation 39, we set *ρ* = 0.623, *ρ*_0_ = 1.6051, and *ρ*_1_ = 2.6455. We generate 10^6^ chaotic data points. The *p*-value from DFT is 0.8472, which indicates that the map exhibits no periodicity.

The algorithm for period measurement using DFT is introduced as follows:

Step 1: Convert 0 and 1 in the sequence $x=\left\{x_{0},x_{1},\cdots \;,x_{n-1}\right\}$ to −1 and 1, which generates a new sequence $x_{B}=\left\{x_{b_{0}},x_{b_{1}},\cdots \;,x_{b_{n-1}}\right\}$. The relationship is given by $x_{b_{i}}=2x_{i}-1$.

Step 2: Perform Fourier transform on the ***x***_*B*_ to generate, $x_{F}=\left\{x_{f_{0}},x_{f_{1}},\cdots \;,x_{f_{n-1}}\right\}$, denoted as $x_{F}=DFT\left(x_{B}\right)$.

Step 3: Calculate $x_{m_{j}}=modulus\left(x_{f_{j}}\right)$, and obtain ***x***_*M*_ = $\left\{x_{m_{0}},x_{m_{1}},\cdots \;,x_{m_{\frac{n}{2}-1}}\right\}$, $0\leq j\leq \frac{n}{2}-1$.

Step 4: Calculate the threshold value $th=\sqrt{\left(\ln\frac{1}{0.05}\right)n}$. Assuming a random hypothesis, 95% of peak heights will not exceed *th*.

Step 5: The number of elements in ***x***_*M*_ exceeding *th* is defined as *N*_0_, while the number not exceeding *th* is defined as *N*_1_.

Step 6: Compute $diff=\frac{2\left(N_{1}-N_{0}\right)}{0.5\sqrt{0.19n}}$.

Step 7: Calculate *p*-value = $erfc\left(\frac{\left|diff\right|}{\sqrt{2}}\right)$, and $erfc\left(x\right)=\frac{2}{\sqrt{\pi }}\int_{x}^{\infty }e^{-t^{2}}dt$.

Map 14: The Tanh-Gauss map is^36^

(Equation 40) $$x_{i+1}=\rho_{0}x_{i}-2\tanh\left(\rho_{1}x_{i}\right)\exp\left(-3{x_{i}}^{2}\right).$$

According to,^36^ set *ρ*_0_ = 0.9, *ρ*_1_ = 5, and initialize *x*_0_ randomly below 1. Figure 5 shows the distribution of numbers from Tanh-Gauss map. The horizontal axis gives outputs; the vertical axis their frequency.

In order to achieve a more uniform distribution of Tanh-Gauss map and reduce complexity, we further derive a simplified version, referred to as the Tanh-exp map:

(Equation 41) $$x_{i+1}=x_{i}-2\tanh\left(2x_{i}\right)\exp\left(-\rho x_{i}\right).$$

Set *ρ* to 1.51. The corresponding histogram is illustrated in Figure 6. Compared to Tanh-Gauss map, Tanh-exp map is more uniform. In Tanh-Gauss map, replace $\exp\left(-3{x_{i}}^{2}\right)$ with exp(−*ρx*_*i*_), which decreases complexity.

Utilizing the DFT periodicity detection algorithm, the *p*-value for Tanh-exp map is determined to be 0.8114, indicating that the Tanh-exp map lacks periodicity. To further analyze its dynamic behavior, we next apply the maximum Lyapunov exponent algorithm^37^ to test Tanh-exp map, with results shown in Figure 7. The Lyapunov exponent is positive for chaotic systems. The modified Tanh-exp map demonstrates chaotic behavior under certain parameters.

The maximum Lyapunov exponent algorithm is as follows:

(1) For the chaotic equation $f\left(x_{n}\right)$, set an iteration count *N*_*L*_ and a small value *d*_0_, and initialize a variable *sum* = 0.
(2) Choose an initial state point *x*_0_ and a nearby point *x*_1_ = *x*_0_ + *d*_0_.
(3) Substitute *x*_0_ and *x*_1_ into the chaotic equation, yielding $y_{0}=f\left(x_{0}\right)$ and $y_{1}=f\left(x_{1}\right)$.
(4) Compute the 1-norm of *y*_0_ and *y*_1_, where $d_{1}=\left\|y_{1}-y_{0}\right\|$.
(5) Solve for *z*_1_ as $z_{1}=y_{0}+\left(d_{0}/d_{1}\right)\left(y_{1}-y_{0}\right)$. Define *x*_1_ = *z*_1_ and *x*_0_ = *y*_0_.
(6) Compute $sum=sum+\log_{e}\left|\frac{d_{1}}{d_{0}}\right|$.
(7) Repeat steps (3) to (6) *N*_*L*_ times, then compute $\lambda =\frac{sum}{N_{L}}=\frac{1}{N_{L}}\overset{N_{L}}{\underset{i=1}{\sum }}{\log_{e}\left|\frac{d_{1}}{d_{0}}\right|}_{i}$.

Map 15: The Bi-sigmoid map is^38^^,^^39^

(Equation 42) $$\left\{\begin{array}{cc} & f\left(v_{i}\right)=\frac{1}{1+e^{-v_{i}/\varepsilon }},\\ & v_{i+1}=\rho_{0}f\left(v_{i}\right)+\rho_{1}v_{i}+a,\\ & x_{i}=\rho_{2}f\left(v_{i+1}\right), \end{array}\right)$$

where *f*(⋅) denotes the standard sigmoid function. Based on the parameters provided in the literature,^38^ Bi-sigmoid map is specifically represented as follows:

(Equation 43) $$\left\{\begin{array}{cc} & f\left(v_{i}\right)=\frac{1}{1+e^{-v_{i}/0.04}},\\ & v_{i+1}=-1.4f\left(v_{i}\right)+0.5v_{i}+1.0,\\ & x_{i}=f\left(v_{i+1}\right). \end{array}\right)$$

However, the numbers generated by the Equation 43 are remarkably uneven, and the corresponding histogram is illustrated in Figure 8.

To overcome the vanishing gradient problem inherent in the traditional sigmoid function at extreme values, and to further broaden the chaotic parameter range of the system, we introduce a reciprocal term and a modulo folding mechanism. Additionally, we upgrade the output stage to a swish activation structure (*x* ⋅sigmoid(*x*)), thereby proposing a novel Swish-modulo map:

(Equation 44) $$\left\{\begin{array}{cc} & v_{i}=\frac{\rho_{0}}{u_{i}}-\rho_{1}u_{i}^{2}+\rho_{2},\\ & u_{i+1}=\;\mathrm{mod}\;\left(v_{i},1\right),\\ & x_{i}=u_{i+1}f\left(u_{i+1}\right),\\ & f\left(u_{i+1}\right)=\frac{1}{1+e^{-u_{i+1}\rho_{3}}}, \end{array}\right)$$

Our goal is to find an algorithm to adjust the parameters *ρ*_0_, *ρ*_1_, *ρ*_2_, and *ρ*_3_ so that *x*_*i*_ is uniformly distributed. However, because the derivative of modulo function is undefined, it is difficult to determine these parameters using gradient descent. Therefore, we use the Bayesian optimization algorithm to set them.

#### Optimization of the 15th chaotic map employing an improved Bayesian approach

The Bayesian optimization algorithm is outlined below. To improve the robustness of the proposed network, the sequences generated by the Swish-modulo map are designed to approach a uniform distribution, which in turn increases the difficulty of network recognition. The objective function for optimization is defined as follows:

(Equation 45) $$f_{\mathrm{obj}}\left(x\right)=-\left\{0.1\sigma^{2}\left(x\right)+0.9|\mu \left(x\right)-0.5|\right\},$$

where $x=\left(x_{0},x_{1},\cdots \;,x_{N_{0}-1}\right)$ represents a 1 × *N*_0_ chaotic sequence. *σ*^2^(***x***) and *μ*(***x***) denote the variance and mean of the sequence.

We employ the Bayesian optimization algorithm to solve the objective function. Figure 9 illustrates the process, and the steps are described as follows:

Step 1: Initialize variables. Set *k* = 0, *j* = 0. Initialize a random matrix

(Equation 46) $$\begin{array}{cc} P_{k} & =\left(\begin{array}{cccc} \rho_{00} & \rho_{01} & \rho_{02} & \rho_{03}\\ \rho_{10} & \rho_{11} & \rho_{12} & \rho_{13}\\ \vdots & \vdots & \vdots & \vdots \\ \rho_{\left(n-1,0\right)} & \rho_{\left(n-1,1\right)} & \rho_{\left(n-1,2\right)} & \rho_{\left(n-1,3\right)} \end{array}\right)\\ & ={\left(\rho_{0},\rho_{1},\cdots \;,\rho_{n-1}\right)}^{T}. \end{array}$$

The dimension of $P_{k}$ is *n* × 4. $\rho_{n-1}=\left\{\rho_{\left(n-1,0\right)},\rho_{\left(n-1,1\right)},\rho_{\left(n-1,2\right)},\rho_{\left(n-1,3\right)}\right\}$.

Step 2: Initialize dataset. Feeding $P_{k}$ row by row into Equation 44 yields the corresponding sequence $X_{k}={\left(x_{0},x_{1},\cdots \;,x_{n-1}\right)}^{T}$. The dimension of ***X***_*k*_ is *n* × *N*_0_. *N*_0_ is the length of vector ***x***_*i*_, where 0 ≤ *i* ≤ *n* − 1. Then, the matrix ***X***_*k*_ is substituted into the objective function *f*_*obj*_(***x***), resulting in $Y_{k}=f_{\mathrm{obj}}\left(X_{k}\right)=\left(y_{0},y_{1},\cdots \;,y_{n-1}\right)$. Assuming the *f*_*obj*_ follows a Gaussian process, the vector ***Y***_*k*_ consequently follows a multivariate Gaussian distribution.

Step 3: Calculate mean and variance. A candidate sampling point $\rho_{j}^{*}$ is randomly selected. Based on the existing dataset $\left(P_{k},Y_{k}\right)$ and the kernel function, the mean $\mu \left(\rho_{j}^{*}\right)$ and variance $\sigma^{2}\left(\rho_{j}^{*}\right)$ are computed utilizing a Gaussian process. Based on the literature ref.^40^ and,^41^ the expressions for mean and variance are as follows:

(Equation 47) $$\left\{\begin{array}{cc} & \sigma_{PP}^{-1}={\left[cov\left(P_{k},P_{k}\right)\right]}^{-1},\\ & \sigma_{\rho^{*}P}=cov\left(\rho_{j}^{*},P_{k}\right),\\ & \mu \left(\rho_{j}^{*}\right)=\sigma_{\rho^{*}P}^{T}\sigma_{PP}^{-1}Y_{k}^{T},\\ & \sigma^{2}\left(\rho_{j}^{*}\right)=cov\left(\rho_{j}^{*},\rho_{j}^{*}\right)-\sigma_{\rho^{*}P}^{T}\sigma_{PP}^{-1}\sigma_{\rho^{*}P}, \end{array}\right)$$

where the superscript *T* denotes the transpose; $\sigma_{\rho^{*}P}\in R^{n\times 1}$ is the covariance vector between $\rho_{j}^{*}$ and $P_{k}$; $cov\left(P_{k},P_{k}\right)\in R^{n\times n}$ is the covariance matrix.

(Equation 48) $$cov\left(P_{k},P_{k}\right)=\left(\begin{array}{ccc} cov\left(\rho_{0},\rho_{0}\right) & \cdots & cov\left(\rho_{0},\rho_{n-1}\right)\\ \vdots & \ddots & \vdots \\ cov\left(\rho_{n-1},\rho_{0}\right) & \cdots & cov\left(\rho_{n-1},\rho_{n-1}\right) \end{array}\right).$$

The cov(⋅) represents the covariance function, also referred to as the kernel function^42^:

(Equation 49) $$cov\left(\rho_{0},\rho_{1}\right)=\frac{2^{1-v}}{\Gamma \left(v\right)}{\left(\frac{\sqrt{2v}}{\lambda }{\left\|\rho_{0}-\rho_{1}\right\|}_{2}\right)}^{v}K_{v}\left(\frac{\sqrt{2v}}{\lambda }{\left\|\rho_{0}-\rho_{1}\right\|}_{2}\right),$$

where *v* and *λ* are hyperparameters; ${\left\|\rho_{0}-\rho_{1}\right\|}_{2}=\sqrt{{\left(\rho_{0}-\rho_{1}\right)}^{T}\left(\rho_{0}-\rho_{1}\right)}$. The expressions for several functions in Equation 49 are as follows:

(Equation 50) $$\left\{\begin{array}{cc} & \Gamma \left(v\right)=\;\int_{0}^{\infty }e^{-x}x^{v-1}dx,\\ & I_{v}\left(x\right)={\left(\frac{x}{2}\right)}^{v}\overset{\infty }{\underset{m=0}{\sum }}\frac{{\left(\frac{x^{2}}{4}\right)}^{m}}{m!\Gamma \left(v+m+1\right)},\\ & K_{v}\left(x\right)=\left(\frac{\pi }{2}\right)\frac{I_{-v}\left(x\right)-I_{v}\left(x\right)}{\sin\left(v\pi \right)}, \end{array}\right)$$

Step 4: Compute acquisition function. By incorporating both the predictive mean and variance, the upper confidence bound (acquisition function) for $\rho_{j}^{*}$ is formulated as follows^43^:

(Equation 51) $$acq\left(\rho_{j}^{*}\right)=\mu \left(\rho_{j}^{*}\right)+\beta \sqrt{\sigma^{2}\left(\rho_{j}^{*}\right)}.$$

In Equation 51, the selection of the hyperparameter *β* directly impacts the quality of the final solution. An improper selection may cause $acq\left(\rho_{j}^{*}\right)$ to converge to a local optimum. Therefore, we propose an adaptive, memory-based acquisition function. Let $\rho_{\mathrm{last}}^{*}$ denote the actual optimal evaluation point selected in the previous outer iteration. For the initial iteration (*k* = 0), $\rho_{\mathrm{last}}^{*}$ is initialized as the evaluation point with the maximum objective value from the initial dataset $P_{k}$. We construct a memory-fused exploration term $vari\left(\rho_{j}^{*}\right)$:

(Equation 52) $$vari\left(\rho_{j}^{*}\right)=0.1\sqrt{\sigma^{2}\left(\rho_{\mathrm{last}}^{*}\right)}+0.9\sqrt{\sigma^{2}\left(\rho_{j}^{*}\right)}.$$

Subsequently, the adaptive acquisition function is formulated as:

(Equation 53) $$acq\left(\rho_{j}^{*}\right)=\mu \left(\rho_{j}^{*}\right)+\frac{\beta \cdot vari\left(\rho_{j}^{*}\right)}{\sqrt{k+1}}.$$

Compared to the standard UCB, Equation 52 dynamically anchors 10% of the exploration weight to the predictive standard deviation of the previously evaluated point, smoothing the variance evaluation and preventing the algorithm from jumping into highly uncertain regions purely randomly. In Equation 53, *β* is a constant scaling factor. Dividing by $\sqrt{k+1}$ (where *k* is the outer iteration counter) strictly controls the exploration decay. As iterations increase, the search shifts from broad exploration to localized refinement. Save $acq\left(\rho_{j}^{*}\right)$ and $\rho_{j}^{*}$ *j* = *j* + 1.

Step 5: Determine next point. Repeat Steps 3 and 4 for *J*_max_ iterations, and determine the next optimal evaluation point $\rho_{n}^{*}$:

(Equation 54) $$\rho_{n}^{*}=\arg\max\left[acq\left(\rho_{j}^{*}\right)\right].$$

Step 6: Update dataset. Substitute the current value $\rho_{n}^{*}$ into Equation 44 to get a sequence. Put this sequence into the objective function *f*_*obj*_(***x***) to get $y_{n}^{*}$. Add $\left(\rho_{n}^{*},y_{n}^{*}\right)$ to the dataset $\left(P_{k},Y_{k}\right)$.

Step 7: Output optimal *ρ*∗. *k* = *k* + 1, *n* = *n* + 1, *j* = 0. If the loop variable *k* reaches the maximum iteration count, output the globally optimal evaluation point from the entire dataset. Otherwise, go to Step 3 and continue iterating.

Based on our enhanced Bayesian search algorithm, the parameters in the chaotic Equation 44 for Swish-modulo map are selected as follows: *ρ*_0_ = 2.3902, *ρ*_1_ = 9.8332, *ρ*_2_ = 8.5699, and *ρ*_3_ = 17.5438. The specific Swish-modulo map is

(Equation 55) $$\left\{\begin{array}{cc} & v_{i}=\frac{2.3902}{u_{i}}-9.8332u_{i}^{2}+8.5699,\\ & u_{i+1}=\;\mathrm{mod}\;\;\left(v_{i},1\right),\\ & x_{i}=u_{i+1}f\left(u_{i+1}\right),\\ & f\left(u_{i+1}\right)=\frac{1}{1+e^{-17.5438u_{i+1}}}, \end{array}\right)$$

The histogram distribution of Swish-modulo map is illustrated in Figure 10.

We also run the DFT periodicity test, yielding a *p*-value of 0.8257 and indicating non-periodicity. We then use the maximum Lyapunov exponent algorithm to evaluate the Swish-modulo map. As shown in Figure 11, the result suggests that the Swish-modulo map exhibits chaotic behavior.

In our experimental tests, the Bernoulli shift map uses *ρ*0 = 1.99 and *ρ*1 = 0.99. The parameter *ρ* in Chebyshev map is set to 4. In the circle map, *ρ*0 = 0 and *ρ*1 = −6. The cubic map has *ρ* = 2.595, and the Fuch map uses *ρ* = 0.8. The Gauss map employs *ρ*0 = 5.9 and *ρ*1 = −0.5. The iterative map, logistic map, PWLC map, sine map, Singer map, and sinusoidal map use parameters *ρ* = 0.7, 4, 0.4, 0.99, 1.07, and 2.3 respectively.

#### Feature extraction driven by the wavelet module

Ingrid Daubechies develops the Symlet wavelet by enhancing the structure based on the dbN wavelet.^44^ Symlet does not have a fixed scale function or a specific wavelet base.^45^ However, the square of the DFT of its scale function coefficients has a definite form with coefficients in^46^

(Equation 56) $$m_{0}\left(\omega \right)=\frac{1}{\sqrt{2}}\overset{2N-1}{\underset{k=0}{\sum }}h_{k}e^{-jk\omega },$$

where *ω* is the angular frequency, *h*_*k*_ is the scale function coefficient.

The square of modulus of the DFT is

(Equation 57) $${\left|m_{0}\left(\omega \right)\right|}^{2}={\left[\cos^{2}\frac{\omega }{2}\right]}^{N}P\left(\sin^{2}\frac{\omega }{2}\right),$$

where $P\left(y\right)=\overset{N-1}{\underset{k=0}{\sum }}C_{k}^{N-1+k}y^{k}$.

The ⊗, ⊕ and ⊙ represent multiplication, addition, and the Hadamard product, respectively. In Figure 12, the conv1 and conv2 indicate one-dimensional convolution operations. Convolution operations can efficiently extract features.^47^ The wavelet we employ is Symlet4, undergoing a first-level decomposition. The discrete wavelet transform of a signal is obtained by applying a series of high-pass and low-pass filters.^48^ The chaos time series ***x***_*t*_ is split into two components after discrete wavelet transformation: the low-frequency approximation ***x***_*ca*_ and the high-frequency details ***x***_*cd*_. These are then processed through two 1D convolution operations to extract features, resulting in ***x***_*ca*_*c*_ and ***x***_*cd*_*c*_ respectively. The parameters for convolution1 and convolution2 are automatically learned during training. The ***x***_*ca*_*c*_ and ***x***_*cd*_*c*_ are transformed into ***x***_*ca*_*cs*_ and ***x***_*cd*_*cs*_ through the sigmoid activation function. For convenience, the sigmoid function is abbreviated as the sig function when writing mathematical formulas. In Figure 12, ***x***_*ca*_*cst*_ and ***x***_*cd*_*cst*_ are represented as

(Equation 58) $$\left\{\begin{array}{cc} & x_{ca\text{\_}cst}=sig\left(x_{ca\text{\_}c}\right)⊙x_{ca}+x_{ca}\\ & x_{cd\text{\_}cst}=sig\left(x_{cd\text{\_}c}\right)⊙x_{cd}+x_{cd} \end{array}\right)$$

In 58, the sig(⋅) function is multiplied by the frequency information ***x***_*ca*_, representing an attention operation. This operation allows ***x***_*ca*_ to retain significant values while disregarding those that are not important. Subsequently, $x_{ca_{c}st}$ and $x_{cd_{c}st}$ undergo a wavelet inverse transform to produce ***x***_*f*_ that incorporates frequency information.

#### Method comparison: Conventional convolution vs. causal operation

Let *n* be the length of the input time series ***x***_*t*_. We apply a one-dimensional convolution to ***x***_*t*_ with a kernel length of 3, using three weights: *w*_0_, *w*_1_, and *w*_2_, as Figure 13 shows. To keep the convolution output ***y*** at length *n*, we pad ***x***_*t*_ with real values at both ends. The first output *y*_0_ is calculated as follows:

(Equation 59) $$y_{0}=\;w_{0}\cdot \left(padding\right)+w_{1}x_{0}+w_{2}x_{1}.$$

The input and output *y*_0_ are misaligned, which is inconsistent with causality. In chaotic inputs, *x*_0_ is generated before *x*_1_, so *y*_0_ should not use *x*_1_ and must depend only on *x*_0_. We suggest an aligned causal method that avoids input padding. Figures 14 and 15 illustrate the computational schematics for *y*_0_ and *y*_1_. With this approach, *y*_0_ and *y*_1_ depend only on previous points. Specifically, the computation of *y*_0_ involves only *x*_0_, yielding *y*_0_ = *w*_0_*x*_0_. Similarly, the computation of *y*_1_ involves only *x*_0_ and *x*_1_, *y*_1_ = *w*_0_*x*_0_ + *w*_1_*x*_1_. This method enables causal alignment and ensures that each output uses only past inputs.

#### Algorithm acceleration: Spectral-inversion-free FFT for efficient cross-correlation

The causal alignment operation is a truncated cross-correlation using only the first *n* results. Direct cross-correlation has time complexity *O*(*n*^2^). Using the FFT reduces the complexity to *O*(*n* log  *n*).^49^ The *X*(*k*) and *W*(*k*) represent the discrete Fourier transforms of *x*(*n*) and *w*(*n*), respectively. For sequences *x*(*n*) and *w*(*n*), their discrete cross-correlation is defined as

(Equation 60) $$\begin{array}{cc} y\left(q\right) & =\overset{N-1}{\underset{m=0}{\sum }}x\left(m\right)w\left(m-q\right)\\ & =\overset{N-1}{\underset{m=0}{\sum }}x\left(m\right)w\left[-\left(q-m\right)\right]\\ & =\frac{1}{N}\overset{N-1}{\underset{k=0}{\sum }}X\left(k\right)W\left(-k\right)e^{-j2\pi qk/\left(2N-1\right)}\\ & =\frac{1}{N}\overset{N-1}{\underset{k=0}{\sum }}Y\left(k\right)e^{-j2\pi qk/\left(2N-1\right)},-\left(N-1\right)\leq q\leq N-1. \end{array}$$

In Equation 60, *k* in *W*(*k*) takes negative values and multiplies *X*(*k*) to produce *Y*(*k*). Based on Equation 60, we propose a spectral-inversion-free FFT-based cross-correlation (SFFT-CC):

(1) Perform an FFT on the sequences *x*(*n*) and *w*(*n*) to obtain *X*(*k*) and *W*(*k*).
(2) Rather than compute *Y*(*k*) = *X*(*k*)*W*(−*k*), we use *Y*(*k*) = *X*(*k*)*W*(*k*). The weight sequence *w*(*n*) can learn and adjust autonomously, so its transform *W*(*k*) is also self-adjusting. Simplify the frequency-domain calculation by removing the *W*(*k*) reversal operation. This approach greatly reduces the need for negative multiplications and subtractions, thereby speeding up computation and simplifying code.
(3) Perform the inverse FFT on *Y*(*k*) to obtain *y*(*n*).
(4) Extract the first *N* values of *y*(*n*) to obtain the final causal alignment result.

### Quantification and statistical analysis

There are no quantification or statistical analyses to include in this study.
